# The transcription factor Dysfusion promotes fold and joint morphogenesis through regulation of Rho1

**DOI:** 10.1371/journal.pgen.1007584

**Published:** 2018-08-06

**Authors:** Sergio Córdoba, Carlos Estella

**Affiliations:** Departamento de Biología Molecular and Centro de Biología Molecular Severo Ochoa, Universidad Autónoma de Madrid (UAM)-CSIC, Madrid, Spain; The University of North Carolina at Chapel Hill, UNITED STATES

## Abstract

The mechanisms that control tissue patterning and cell behavior are extensively studied separately, but much less is known about how these two processes are coordinated. Here we show that the *Drosophila* transcription factor Dysfusion (Dysf) directs leg epithelial folding and joint formation through the regulation of Rho1 activity. We found that Dysf-induced Rho1 activity promotes apical constriction specifically in folding epithelial cells. Here we show that downregulation of Rho1 or its downstream effectors cause defects in fold and joint formation. In addition, Rho1 and its effectors are sufficient to induce the formation of epithelial folds when misexpressed in a flat epithelium. Furthermore, as apoptotic cells can actively control tissue remodeling, we analyzed the role of cell death in the formation of tarsal folds and its relation to Rho1 activity. Surprisingly, we found no defects in this process when apoptosis is inhibited. Our results highlight the coordination between a patterning transcription factor and the cellular processes that cause the cell shape changes necessary to sculpt a flat epithelium into a three dimensional structure.

## Author summary

Epithelial morphogenesis drives the formation of organs and the acquisition of body shape. Changes in cell behavior such as cell proliferation, cell shape or apoptosis contribute to the remodeling of the epithelia from a simple layer to a three dimensional structure. These changes have to be precisely regulated by an underlying patterning network to control the final shape of an organ. However, how these two processes are coordinated is mostly unknown. In this work we use the formation of the fly leg joints as a model to study how Dysfusion (Dysf), a patterning transcription factor, regulates the cellular mechanisms that form the folds in the leg discs epithelium. We have found that *dysf* modulates the localization and activity of Rho1, a key regulator of the acto-myosin cytoskeleton, to drive cell apical constriction and epithelial folding in the leg disc. Furthermore, in this work we provide proof of the direct requirements of Rho1 and its downstream effectors in fold and joint formation. We conclude that Dysf-regulated Rho1 activity controls the cell shape changes that sculpt leg joints.

## Introduction

Epithelial morphogenesis plays a central role in the formation of organs and the acquisition of body shape. During development, epithelial tissues undergo extensive remodeling through changes in cell proliferation, apoptosis, cell-cell interactions and cell shape (reviewed in [1]). These changes allow the formation of complex three-dimensional structures from simple epithelial sheets. Epithelial morphogenesis requires the activation of specific gene expression programs and their implementation in terms of collective cell behavior. Although the mechanisms that control tissue patterning and cell behavior are extensively studied separately, much less is known about how these two processes are coordinated.

The development of imaginal discs in *Drosophila* is a convenient model to study epithelial morphogenesis. Imaginal discs are epithelial tissues formed by a pseudostratified monolayer of cells, which will form adult cuticular structures such as the wing or the leg [2, 3]. During the larval stages, imaginal discs grow and are subdivided into different domains of gene expression [4, 5]. During metamorphosis, a pulse of the steroid hormone ecdysone triggers the beginning of pupation, the process through which imaginal discs are shaped into their correspondent three-dimensional adult structures [6, 7].

In this work, we use the development of the leg joints of *Drosophila* as a model to elucidate the relationship between positional information and the cellular mechanisms that shape the tissue. The joints are flexible structures that subdivide the leg in 10 segments, allowing locomotion. The different segments that comprise the adult leg are genetically determined during larval development, when the leg disc is progressively subdivided by the nested expression of transcription factors that confer positional identity along the proximo-distal (P-D) axis (reviewed by [8, 9]). This positional information is necessary for the expression, in bands of cells, of the Notch ligands, Delta (Dl) and Serrate (Ser) [10, 11]. Notch activation at the distal border between each presumptive segment of the leg is necessary to form all leg joints [12–14]. The five tarsi, the distal-most segments of the leg, are separated by four ball-and-socket shaped joints that are morphologically and evolutionarily different from the “true” or proximal joints [15–20]. Tarsal joint formation is specifically regulated by *dysfusion* (*dysf*), a gene directly regulated by Notch that encodes a bHLH-PAS transcription factor [21].

**Fig 1:**
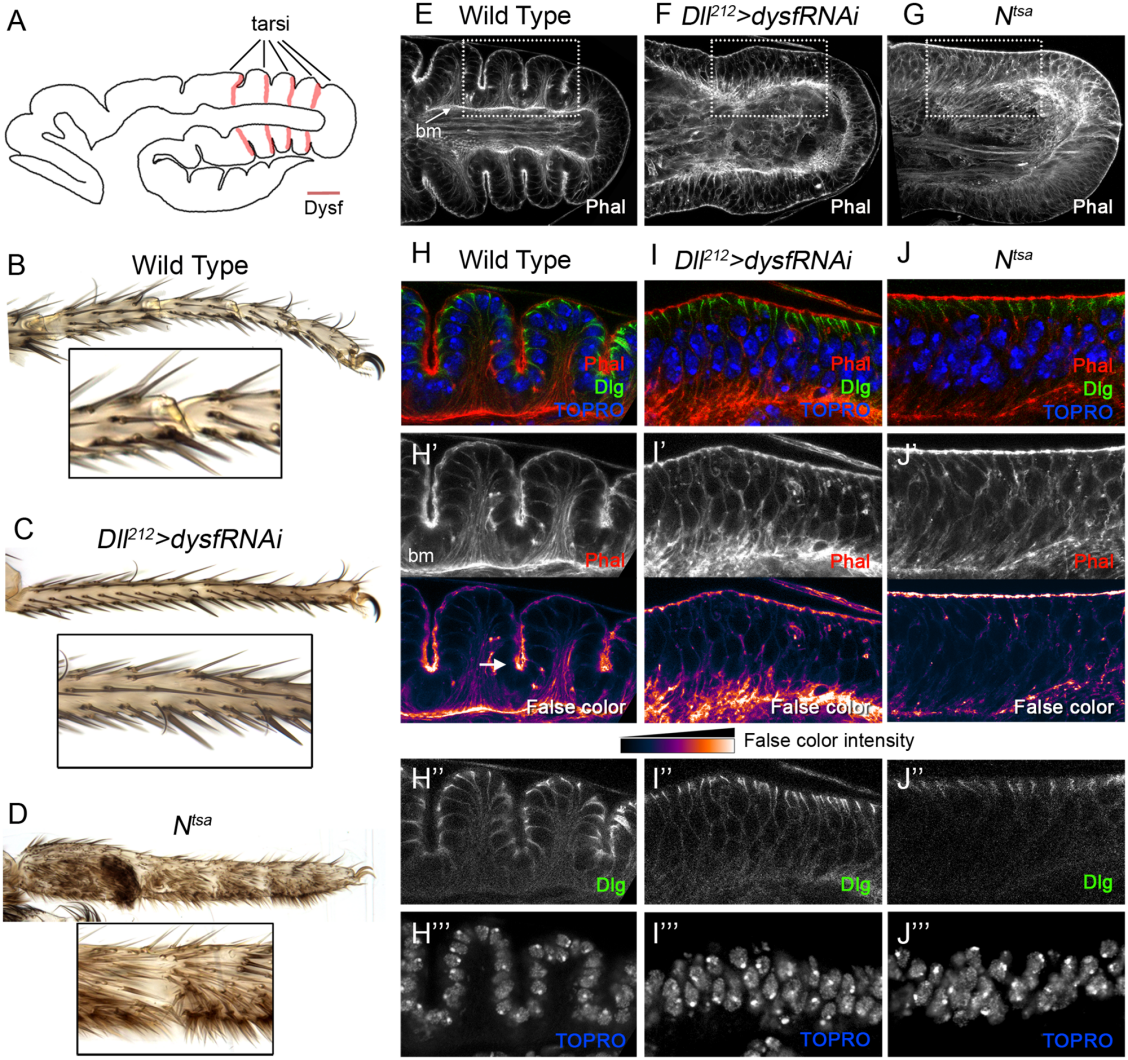
Fold formation is impaired in *dysf* loss of function. (A) Schematic representation of a prepupal leg disc, where the location of the presumptive five tarsi is indicated and *dysf* expression is marked in pink. In this figure and onwards, Proximal is to the left and Distal to the right. (B-D) Tarsal region of wild type (B), *Dll*^*212*^*>dysfRNAi* (C) and *N*^*tsa*^ (D) adult legs. An amplification of the joint region is shown below each leg. Note the complete absence of tarsal joints in C and D. (E-G) Tarsal region of prepupal leg discs (sagittal section of the epithelium) of wild type (E), *Dll*^*212*^*>dysfRNAi* (F) and *N*^*tsa*^ (G) stained with Phalloidin (Phal, in grey channel) to visualize F-actin cytoskeleton. Basement membrane, bm. (H-J) Higher magnification of the prepupal leg disc epithelium from the genotypes presented above (dotted squares). Phal staining is in red in H-J (separate channel and in false color to enhance contrast in H’-J’). The arrow in H’ points to a fold, where higher intensity of F-actin is present. Compare H’ with I’ and J’ where no fold is formed and the intensity of apical F-actin remains homogeneous throughout the epithelium. The baso-lateral protein Discs-large (Dlg) is in green in H-J (separate channel in H”-J”) and the nuclear marker TOPRO is in blue in H-J (separate channel in H”’-J”’).

The formation of tarsal joints is first evident during prepupal development as four deep folds in the epithelium perpendicular to the P-D axis (Fig 1A and 1E). Similarly to other morphogenetic processes ([22–24] and reviewed in [25]), coordinated cell apical constriction is observed at the folding of the epithelium (the presumptive joints) [18, 26, 27]. Apical constriction is a common cellular mechanism by which a cell, or group of cells, reduce their apical domain causing a cell shape change that may result in tissue folding [25, 28]. Despite variations on the different developmental contexts, there is a limited core of elements required for apical constriction: 1) the activation of acto-myosin contractility, which provides the force required to generate the contraction of the cell apex and 2) adhesion proteins that couple the actin cytoskeleton to the adherens junctions, that allows force transmission to neighboring cells [25] [29]. The contractility of the cell during this process is regulated by the activity of the small RhoGTPases, especially by Rho1 [25, 30]. Rho1 activity is finely tuned by Rho guanine nucleotide exchange factors (RhoGEFs) and Rho GTPase activating proteins (RhoGAPs), which respectively activate and inhibit Rho1.

Active Rho1 recruits different targets, the most prominent being Rho kinase (Rok), that in turn activates non-muscle Myosin II (MyoII) [30–33]. MyoII is a multimeric protein composed of two heavy chains, encoded in *Drosophila* by the gene *zipper* (*zip*) that binds actin and provides the motor activity for acto-myosin contraction, two regulatory light chains, encoded by *spaghetti squash* (*sqh*), that are activated through phosphorylation by Rok and two essential light chains encoded by *Mlc-c* [31, 34, 35]. Another kinase, the Death-associated protein kinase related (Drak), has redundant roles with Rok in the phosphorylation of Sqh, and its function is required only when Rok activity is impaired [36].

An additional target of Rho1 is Diaphanous (Dia), the only representative of the Formins family in *Drosophila*. Dia acts coordinating acto-myosin contractility and F-actin nucleation at the adherens junctions during cell shape changes, and is needed for apical constriction in several developmental contexts [23, 37].

Two morphogenetic mechanisms have been implicated in controlling the cell shape changes occurring during fold formation at the presumptive tarsal joints. One is the localized cell death at the tarsal folds, caused by the activation of the pro-apoptotic genes *reaper* (*rpr*) and *head involution defective* (*hid*) [17, 21]. Recently, apoptosis has been proposed as the initial trigger that generates mechanical force driving the formation of the tarsal folds [26]. In this model, dying cells cause transient folds in the epithelium that are later stabilized into permanent folds by the presence of MyoII, leading to apical constriction and consequent epithelial folding [26]. Second, a number of RhoGEFs and RhoGAPs are expressed and required specifically at the tarsal joints [38, 39]. Interestingly, *dysf* transcriptionally regulates both, the pro-apoptotic genes *rpr* and *hid*, and the RhoGTPase regulators *RhoGEF2* and *RhoGAP71E* [21]. Yet, it is not clear whether the activity of Rho GTPases, which have a fundamental role in various models of apical constriction, is required in the context of tarsal fold formation. Nevertheless the extent to which RhoGTPases contribute to apical constriction at the tarsal folds and the relationship between cell death and cell shape changes remains largely unknown.

In this work, we found that Dysf regulates Rho1 activity at the tarsal epithelial folds and that Rho1 function is required for fold and adult joint formation. In addition we analyzed the requirement of the Rho1 effectors during joint development and their ability to induce folds. Unexpectedly, no defects were found when cell death was inhibited in the leg either in Rho1 activity or in joint formation. We propose a model where the restricted activation of the Notch pathway in concentric rings induces Rho1 activity through *dysf* to mediate apical constriction and tarsal joint formation.

## Results

### *dysfusion* loss of function impairs apical constriction at the presumptive leg joints

The formation of the adult tarsal joints is prefigured in the prepupal leg imaginal discs as deep epithelial constrictions that are positioned distally to each Notch activation domain [12, 21]. At the tarsal domain, four transversal folds are clearly observed that divide it in five tarsal segments (Fig 1A and 1B). In order to form these structures, cells undergo coordinated apical constriction, causing the physical separation of each tarsal segment by a pronounced fold in the epithelium [27] (Fig 1E and S1 Fig). Apical constriction of these cells is characterized by an accumulation of F-actin at their apices and a prominent shortening along their apico-basal axis (Fig 1H). Interestingly, the basal membrane remains flat compared to the apical side. In addition, the leg epithelium changes from pseudostratified to simple, as observed by the regular alignment of the cell nuclei (Fig 1H) [39]. To better understand the cellular processes that drive fold morphogenesis we compared the prepupal leg epithelium of wild type legs with legs where the formation of the tarsal folds is inhibited in two different ways. First, we downregulated the function of *dysf*, a Notch-induced target gene expressed in four concentric rings that is completely necessary for both, the presence of the folds in the prepupal disc and the formation of the adult tarsal joints (Fig 1A and 1C) [21]. Second, we used a temperature-sensitive allele of *Notch* (*N*^*tsa*^) that causes an absence of tarsal joints when animals are switched to the restrictive temperature (Fig 1D, see Material and methods) [40].

When *dysf* activity is knocked down in the distal domain of the leg (*Dll*^*212*^*>dysfRNAi*) or in *dysf* mutants [21], we observed the flattening of the prepupal epithelium, no apical constriction and a total absence of adult tarsal joints (Fig 1C and 1F and S1 Fig). While in control legs high levels of F-actin accumulated at the apical domain of the cells that form the fold, in *Dll*^*212*^*>dysfRNAi* prepupal leg discs, apical F-actin remains evenly distributed along the leg disc epithelium, and no folds are formed (compare Fig 1I with Fig 1H). It is important to note leg segmentation is not disrupted in these mutant conditions as the rings of Ser/Dl and *E(spl)mβ* (a target of the Notch pathway) are correctly positioned (S2 Fig and [21]). This difference in F-actin distribution is not caused by defects in apico-basal polarity of the putative fold-forming cells as the baso-lateral marker, Discs large (Dlg), is correctly located (compare Fig 1I and 1H). Moreover, the characteristic remodeling of the epithelium from pseudostratified to simple is not observed in *dysf* knockdown prepupal leg discs (compare Fig 1I and 1H). Interestingly, this failure on nuclei alignment was also described for RhoGAP68F knockdown prepupal leg discs that also presented defects in fold formation [39]. The same cellular phenotypes of apical F-actin distribution, absence of tarsal folds and nuclear arrangement described for *Dll*^*212*^*>dysfRNAi* were observed in *N*^*tsa*^ prepupal leg discs (Fig 1G and 1J).

### Localization of Rho1 and MyoII during joint formation

**Fig 2:**
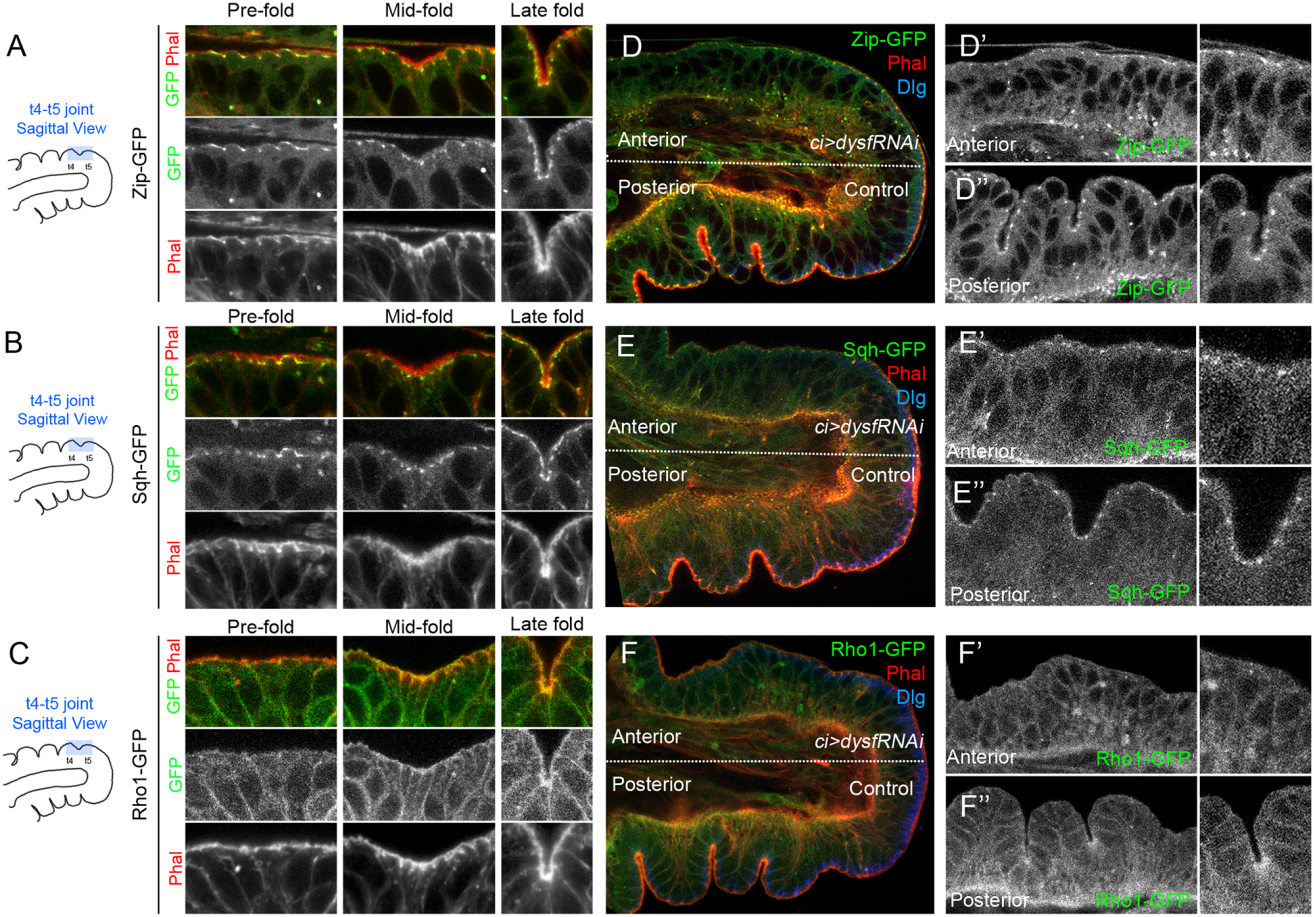
Rho1 and MyoII localization during fold formation and in Dysf knockdown prepupal legs. (A-C) Time course imaging of the apical region (sagittal view, schematic representation to the left) of the t4-t5 tarsal fold at the Pre-fold stage, Mid-fold stage and Late fold stage of *zip-GFP* (A), *sqh*^*AX3*^*; sqh-GFP* (B) and *Rho1-GFP* (C) prepupal leg discs. Zip-GFP, Sqh-GFP and Rho1-GFP are in green in A-C, and Phal is in red. Separate grey channels for GFP and Phal are presented below. Note that Zip-GFP and Sqh-GFP remains localized at subapical puncta as fold formation proceeds, while Rho1-GFP is accumulated in the apical region from Mid-fold onwards, resembling F-actin accumulation. (D-F) Prepupal leg discs where Dysf levels are downregulated in the anterior compartment (*ci>dysfRNAi*). Zip-GFP, Sqh-GFP and Rho1-GFP are in green in D-F. Anterior compartments are magnified in D’, E’ and F’ and posterior in D”, E” and F”, where GFP is shown in grey channel. A close zoom of a single tarsal joint is at the right of each panel. Note that the folds are not formed in the anterior compartment where *dysf* expression is downregulated. Phal is in red and Dlg is in blue.

As apical constriction is mediated by acto-myosin contraction regulated by Rho GTPases [28] we decided to follow Rho1 and MyoII localization during joint formation using GFP-tagged forms of Rho1, Sqh (MyoII regulatory light chain) and Zip (MyoII heavy chain). Zip and Sqh are located subapically at the level of the adherens junctions along the leg disc epithelium and no changes in localization are observed in fold-forming cells (Fig 2A and 2B). Conversely, Rho1 is specifically enriched in the apical domain of invaginating cells as fold progresses, and co-localizes with the accumulation of F-actin (Fig 2C).

Next, we tested whether the lack of F-actin accumulation and apical constriction observed in *dysf* knockdown prepupal legs is caused by mislocalization of Rho1 or MyoII. Downregulation of Dysf in the anterior compartment under the control of the *cubitus interruptus* (*ci*) *Gal4* driver (*ci>dysfRNAi*) efficiently abolished tarsal fold and joint formation (Fig 2D–2F). Importantly, while Sqh or Zip localization is not altered when *dysf* activity is knocked down, we observed a lack of apical Rho1-GFP accumulation in these cells when compared to the control compartment (Fig 2D–2F). These results suggest that defects in F-actin and apical constriction may be due to Rho1 accumulation defects and not to MyoII mislocalization.

### Rho1 activity is enhanced at the tarsal epithelial folds and depends on *dysf*

**Fig 3:**
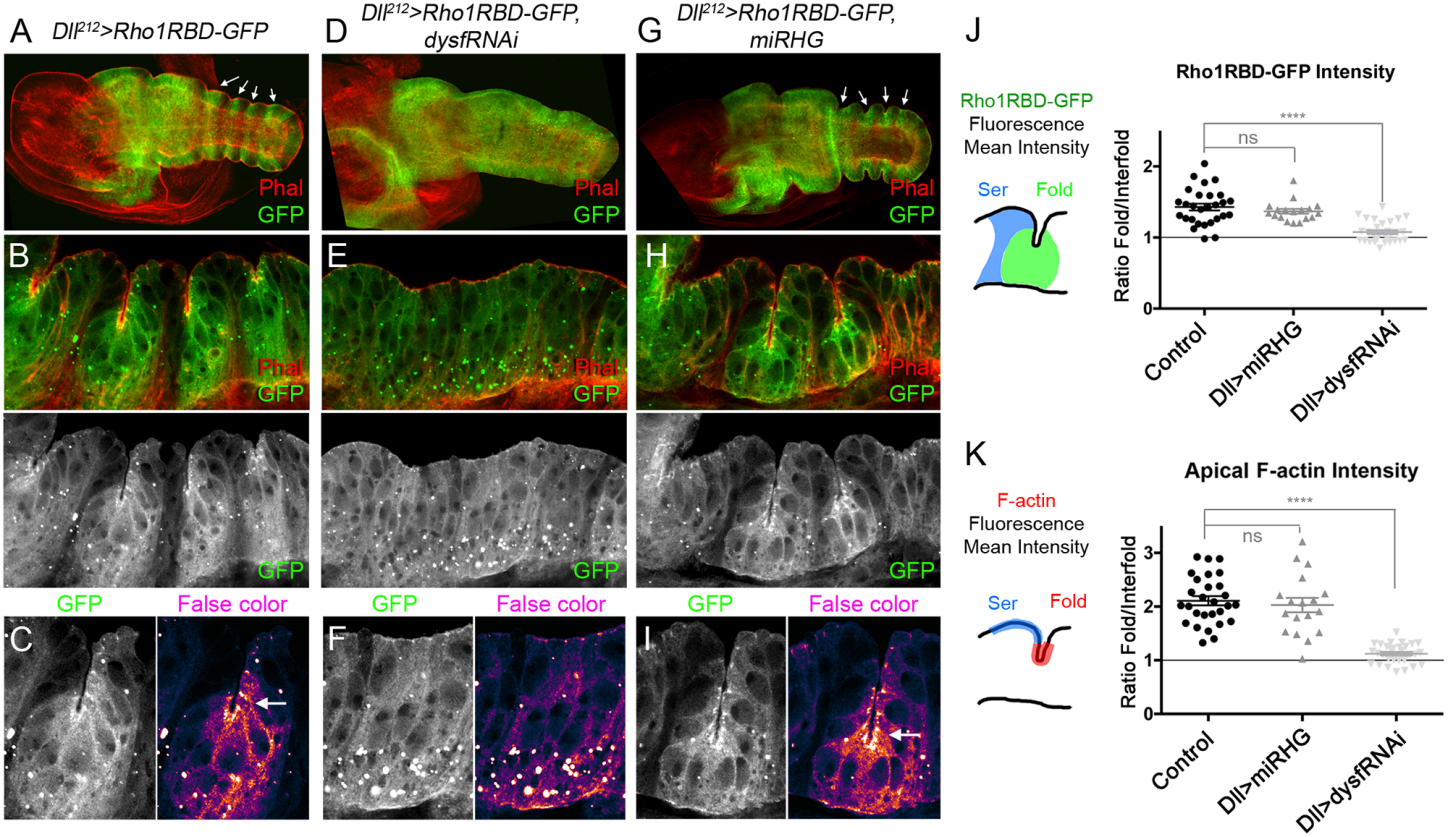
Rho1 activity pattern is altered in *dysf* loss of function. (A-I) 3 hrs APF leg discs of the following genotypes: *Dll*^*212*^*>Rho1RBD-GFP* (A-C), *Dll*^*212*^*>Rho1RBD-GFP*, *dysfRNAi* (D-F) and *Dll*^*212*^*>Rho1RBD-GFP*, *miRHG* (G-I). Note that the striped pattern of Rho1 activity (arrows) is lost in D. Phal is in red and Rho1RBD-GFP in green. (B, E and H) Close up views of the distal leg epithelium (sagittal section) of the genotypes above. Regions of enhanced GFP levels are seen around the folds in B and H that are separated by regions of lower GFP levels in the interfold regions. This pattern is lost in E, where GFP levels remain homogeneous throughout the epithelium. Phal is in red and Rho1RBD-GFP in green and in separate channel below. (C, F and I) Magnification of a fold or putative fold region of the genotypes above, showing separate channel for GFP to the left and false color to enhance contrast to the right. GFP levels are accumulated apically in C and I in the cells that are undergoing apical constriction (arrow), while in F GFP is evenly distributed across the cells. (J and K) Ratio of fluorescence levels of Rho1RBD-GFP (mean intensity) (J) and apical F-actin (mean intensity) (K) within fold and interfold domains (Ser positive cells) for the previous genotypes (see also S2 Fig). In both cases, a ratio of 1 would imply the same levels in fold and interfold domains, while any increment over 1 means higher levels in the fold *vs* interfold domain. In control *Dll*^*212*^*>Rho1RBD-GFP* and *Dll*^*212*^*>Rho1RBD-GFP*, *miRHG*, the fold/interfold ratio for GFP and apical F-actin intensity is close to 1.5 and 2, respectively. However, when Dysf is knocked down, the fold/interfold ratio for GFP and apical F-actin intensity drops to close to 1. t2-t3 and t3-t4 folds were used for quantification (control n = 28 joints; *miRHG* n = 18 joints and *dysfRNAi* n = 30 joints for both measurements). ****p<0.0001, with Student’s t test, indicating a significant difference from control. ns, non-significant. Error bars represent SEM.

Both, the regulation of Rho GTPases activity and apoptosis have been implicated in tarsal joint formation [17, 26, 38]. Dysf regulates the expression of the Rho1 activity regulators, *RhoGEF2* and *RhoGAP71E* and of the pro-apoptotic genes *rpr* and *hid* [21]. However, the specific role of the Rho1 GTPase during fold and joint formation and the functional relationship between Rho1 and cell death in this process are unknown. Therefore, we decided to monitor Rho1 activity during leg epithelium morphogenesis with a GFP-based sensor that can be expressed *in vivo* using the Gal4/UAS system [41]. This construct, here termed Rho1RBD-GFP (Rho1 Rho Binding Domain-GFP), recognizes the GTP-bound (active) form of Rho1. Consequently, GFP will be accumulated preferentially in regions where Rho1 is active [41]. In control *Dll*^*212*^*>Rho1RBD-GFP* prepupal leg discs (dissected 3 hrs after puparium formation, APF), GFP is detected in the *Dll* domain at higher levels in four bands of cells that encompasses the tarsal folds, while the regions between folds show lower levels of GFP (Fig 3A–3C). Interestingly, high levels of GFP appear around the apical domain and at the level of the adherens junctions of the cells that undergo apical constriction to form the folds, indicating a concentration of active Rho1 in that region (Fig 3B and 3C and S3 Fig). Consistently, this increase of Rho1 activity occurs in the same cells that accumulate Rho1-GFP at their apical regions during fold formation (see S3 Fig and compare with Fig 2C). Also, GFP is detected at high levels forming small clusters that may correspond to trafficking vesicles (Fig 3B and 3C).

In contrast, in *Dll*^*212*^>*Rho1RBD-GFP*, *dysfRNAi* prepupal leg discs (3 hrs APF), GFP levels appear more homogeneously distributed along the distal region of the leg disc, and no clear bands of GFP expression were detected (Fig 3D–3F). Furthermore, the characteristic GFP apical accumulation described in control discs was not observed, while high levels were detected in small clusters that localize preferentially toward the basal half of the cells (Fig 3E and detail in Fig 3F). To analyze these results in more detail we quantified the relative enrichment of GFP signal and F-actin staining in fold cells compared to proximal interfold cells (identified by Ser antibody staining, see S2 Fig). The fold/interfold signal ratio for GFP and F-actin is close to 1.5 and 2, respectively in control legs, indicating an enrichment of both signals in the fold with respect to the interfold region. These ratios drop to almost 1 in *Dll*^*212*^*>dysfRNAi* legs (Fig 3J and 3K). Similar phenotypes were observed in *N*^*tsa*^ mutant prepupal leg discs (S4 Fig), where the distribution of Rho1RBD-GFP is comparable to that observed in *Dll*^*212*^*>dysfRNAi* legs.

It has been proposed that cell death provides the initial force driving cell reorganization and tissue folding during tarsal joint formation [26]. We reasoned that localized apoptotic cells in the presumptive fold region could activate Rho1, in turn leading to increased MyoII contractility to cause the apical constriction and the formation of a stable fold. To test this hypothesis, we expressed an UAS transgene, which generates miRNAs that simultaneously inhibit the pro-apoptotic genes *rpr*, *hid*, and *grim* (UAS*-miRHG*), and almost completely abolish cell death (quantified in S5 Fig) [42]. Both, F-actin accumulation and Rho1 activity pattern were indistinguishable from control prepupal legs when cell death was inhibited in the *Dll* domain (*Dll*^*212*^*>Rho1RBD-GFP*, *miRHG*) (Fig 3G–3I and quantified in Fig 3J and 3K). Surprisingly, both the folds in the prepupal leg disc and the corresponding tarsal joints in the adult leg were correctly formed (Fig 3G–3I and S5 Fig). To confirm these results we blocked cell death at three additional levels of the apoptotic pathway (S5 Fig). First, we ectopically expressed the *Death-associated inhibitor of apoptosis 1* (*Diap1*) gene in the posterior compartment and in the *Dll* domain. Although a strong reduction of cell death was observed, no joint defects were recovered (S5 Fig). Second, the lack of a joint phenotype in the absence of cell death was confirmed in null mutants for the initiator caspase *dronc* and the combination of *Dll*^*212*^*>miRHG* in a *dronc* mutant background (S5 Fig). Third, we ectopically expressed the baculovirus protein p35 in the *Dll* domain, that block the activity of the executioner caspases [43]. In this case, only minimal defects were observed in joint formation, with approx. 25% of legs presenting 1 or 2 defective joints (S5 Fig).

Taken together, our results suggest that *dysf* regulates Rho1 activity in the tarsal folds, while cell death inhibition does not cause defects in Rho1 activity, fold or joint formation.

### Rho1 is essential for epithelial fold and tarsal joint formation

**Fig 4:**
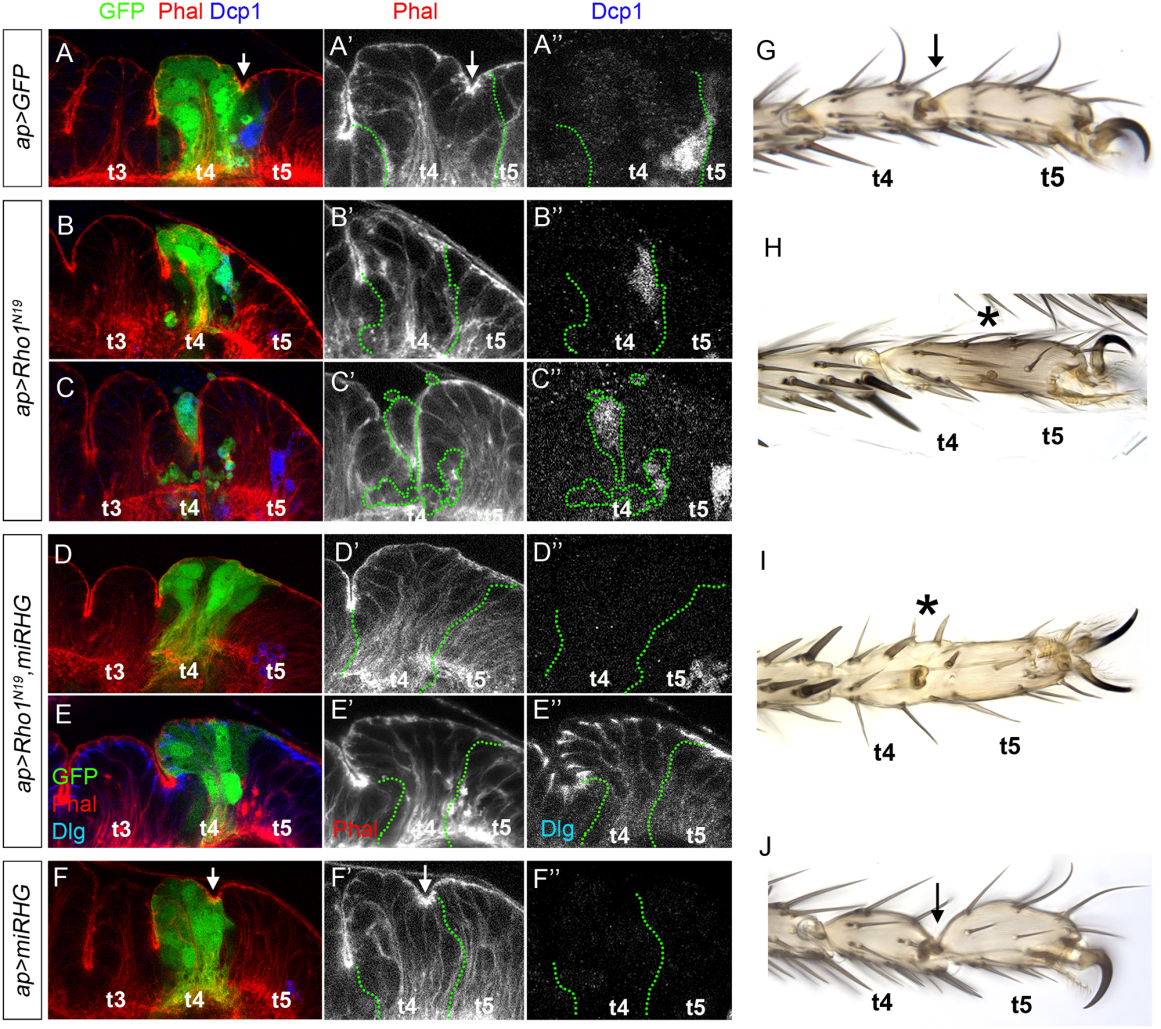
Rho1 activity is necessary for fold and joint formation. (A-F) Distal region of 3 hrs APF leg discs showing the *ap* domain that encompasses the fold between the fourth and fifth tarsal segments (GFP, green). Right panels show a detail of the *ap* domain marked by green dotted lines and the t4-t5 fold (arrows). In A-F Phal is in red and separate channel in A’-F’ and Dcp1 is in blue in A-F and separate channel in A”-F”. Dlg is in blue in E and separate channel in E”. The activity of the *ap-Gal4* driver was restricted to 24 hrs before dissection using the *tubulin-Gal80*^*ts*^ system, except in A and F that was continuously active (see Materials and methods). (A) *ap>GFP* control disc. Note the t4-t5 epithelial fold within the *ap* domain. (B, C) *ap>Rho1*^*N19*^. B and C correspond to opposite sides of the same leg disc. The t4-t5 fold is lost in B, while the epithelial integrity is severely altered in C. (D, E) *ap>Rho1*^*N19*^, *miRHG*. The t4-t5 fold is lost while the epithelial integrity is not compromised as visualized by Dlg staining. (F) *ap>miRHG*. The t4-t5 fold is formed as in the control. (G-J) Adult legs of *ap>GFP* (G), *ap>Rho1*^*N19*^ (H), *ap>Rho1*^*N19*^, *miRHG* (I) and *ap>miRHG* (J). The activity of the *ap-Gal4* driver was restricted to 48 hrs with the *tubulin-Gal80*^*ts*^ system except in G and J that was continuously active (see Materials and methods). In H and I the t4-t5 joint is lost, despite some indentations in the cuticle could still be seen. Note that in *ap>miRHG* legs (J) the fourth and fifth tarsi are slightly shorter and wider compared to the control (G). Arrows indicate formation of the joint between t4 and t5 while asterisks indicate its loss.

To study the specific role of Rho1 in the formation of epithelial folds we knocked down Rho1 activity by expressing a dominant negative form, UAS-*Rho1*^*N19*^ [44], in the *apterous* (*ap*) domain that encompasses the t4-t5 tarsal fold. To properly compare similar age legs, prepupae were synchronized and dissected 3 hrs APF. In wild type prepupal leg discs, the fold that separates t4 and t5 segments is already formed and is readily associated with apoptotic cells (Fig 4A). As prolonged blockage of Rho1 activity is lethal, we restricted *ap*-*Gal4* activity for 24 hrs prior to dissection using the *tubulin-Gal80*^*ts*^ system (see Materials and methods). In these prepupal leg discs, the formation of the t4-t5 fold was abolished (Fig 4B). However, the integrity of the epithelium in some cases was severely compromised, as observed by cell delamination and an increase of apoptosis in the *ap* domain, making difficult the interpretation of the phenotype (compare Fig 4B and 4C with Fig 4A). As Rho1 GTPase operates upstream of the JNK pathway directing apoptosis, the expression of either an activated or dominant negative version of Rho1 increases cell death (our results and [45, 46]). To avoid the deleterious effects that increased cell death may generate, we expressed UAS-*miRHG* along with UAS-*Rho1*^*N1*9^ for 24 hrs before dissection to efficiently inhibit apoptosis. In this case (*ap>Rho1*^*N19*^, *miRHG*), no apical F-actin accumulation was observed and the formation of the t4-t5 fold was also inhibited, while the apico-basal integrity of the *ap-Gal4* domain, as visualized by Dlg staining, was not compromised (Fig 4D and 4E). Consistently with our previous results, the elimination of cell death in the *ap* domain using the UAS-*miRHG* did not have any effect on fold formation (Fig 4F).

To explore the consequences of Rho1 knockdown on adult joint formation, we restricted *ap>Rho1*^*N19*^ expression through late third instar larval stage and early pupation using the *tubulin-Gal80*^*ts*^ system, and then letting the animals recover until pharate (see Materials and methods). A range of phenotypes was observed from complete loss of the t4-t5 joint to indentations that may correspond with incomplete joint formation (Fig 4H). When cell death was simultaneously inhibited (*ap>Rho1*^*N19*^, *miRHG*) the same phenotypes were observed (Fig 4I). As described above, adult legs from animals expressing the UAS-*miRHG* (*ap>miRHG*) for the entire development correctly formed the t4-t5 joints (compare Fig 4J with 4G).

In summary, knockdown of Rho1 activity causes defects in tissue morphology, including defects in apical F-actin accumulation, fold formation and impairment of adult joint development.

### Analysis of Rho1 effectors requirements during joint formation

**Fig 5:**
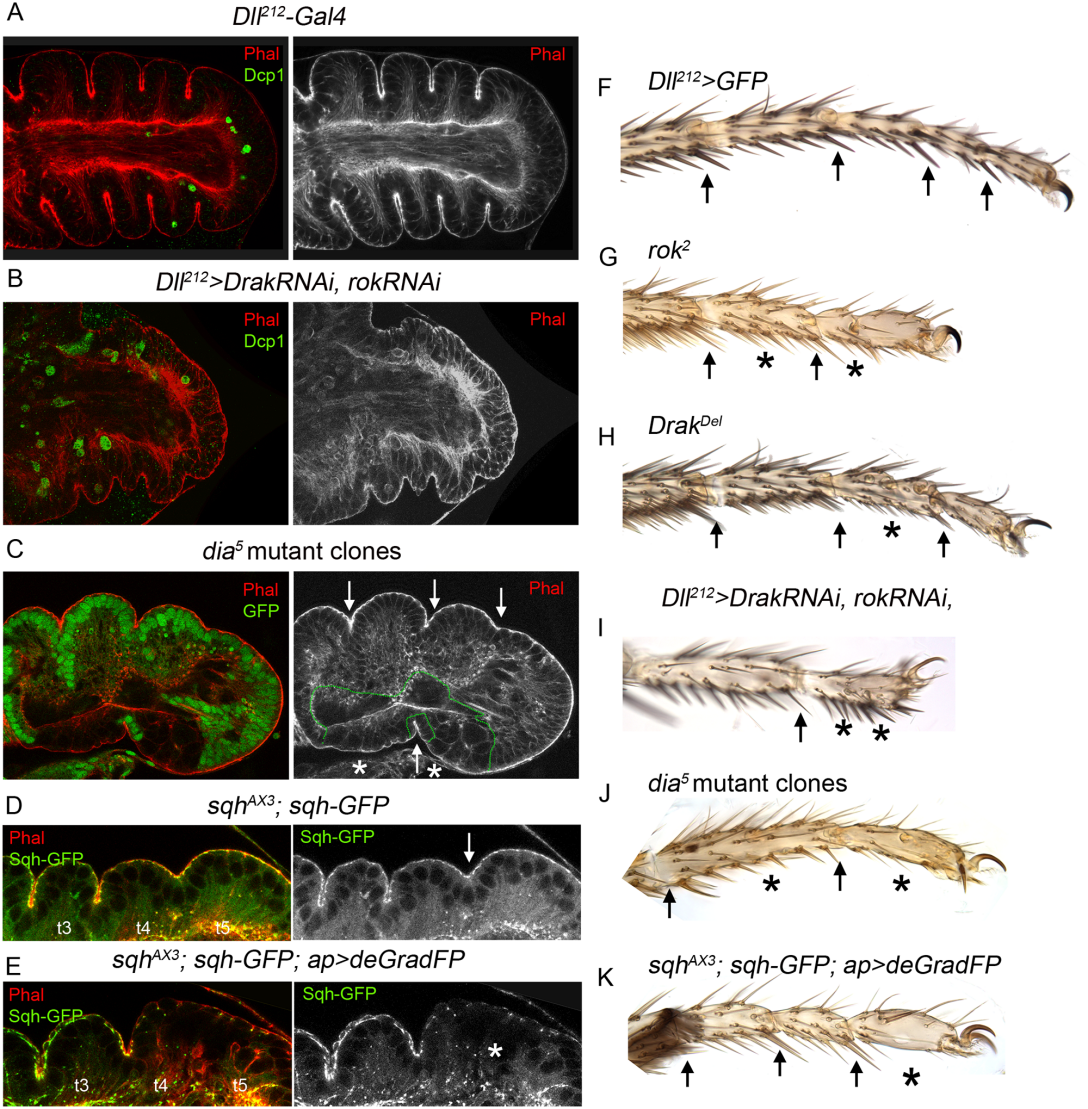
Downregulation of Rho1 effectors cause defects in fold and joint formation. (A, B) Prepupal leg discs (dated 3 hrs APF) of a *Dll*^*212*^*-Gal4* control (A) and *Dll*^*212*^*> DrakRNAi*, *rokRNAi* (B). Phal is in red and separate channel to the right and Dcp1 is in green. Compare the normal formation of the tarsal folds in A with the defective folds and overall tarsal size reduction in B. (C) *dia*^*5*^ mutant clones generated in a *Minute* background marked by the loss of GFP (green and dotted line in the right panel), cause loss of folds (asterisks). Correct fold formation is indicated with an arrow. Phal is in red and separate channel to the right. (D, E) Prepupal leg discs mutant for *sqh* in which Sqh-GFP (green and separate channel to the right) is expressed under the control of the endogenous *sqh* promoter (D). In E, the construct UAS-*deGradFP*, which targets GFP for degradation, is expressed under the *ap-Gal4* driver for 24 hrs with the *tubulin-Gal80*^*ts*^ in a *sqh*^*AX3*^ mutant background, thus depleting Sqh protein in this domain. Note the failure in fold formation between t4 and t5 in E (asterisk). Phal is in red. (F-K) Tarsal region of adult legs of the following genotypes: *Dll*^*212*^*>GFP* (F), *yw rok*^*2*^ *FRT*^*19A*^/*ubi-GFP M(1)osp FRT*^*19A*^*; Dll*^*212*^*>flp* (G), *Drak*^*Del*^ mutant (H), *Dll*^*212*^*>DrakRNAi*, *rokRNAi* (I), *yw hsflp; dia*^*5*^ *FRT*^*40*^*/ubiGFP M(2)z FRT*^*40*^ (J) and adult leg of the experiment in E (K). Arrows indicate correctly formed joints and asterisks point out absence or defective joint. See Material and methods for details.

Rok and Drak are Rho1 downstream targets that have overlapping functions in Sqh phosphorylation, acto-myosin contractility and cytoskeleton remodeling [33, 36]. We have studied the specific contribution of each effector to tarsal leg joint formation. Legs that are completely mutant for *rok* (see Material and methods) are shorter and present a consistent mild defect in joint formation (~80% of the legs lacking one or two joints) (Fig 5G and quantified in S6 Fig). *Drak* mutants are viable and have smaller wings and twisted legs [36], however, the four tarsal joints are present in ~95% of the legs (Fig 5H and S6 Fig). As double mutant clones for *rok* and *Drak* were small and hardly recovered even when cell death was inhibited (S6 Fig), we decided to use RNAi mediated depletion of Rok and Drak. We used the *Dll*^*212*^-*Gal4* line that by its own has a minimal leg phenotype, causing defects in the formation of one tarsal joint in less than 8% of the legs (Fig 5F and S6 Fig) (see Materials and methods). As a positive control, more than 95% of *Dll*^*212*^*>dysfRNAi* legs lost all the tarsal joints (S6 Fig). When both Rok and Drak were simultaneously downregulated in the *Dll* domain with specific RNAi lines, we found stronger phenotypes than using each RNAi line independently (Fig 5I and S6 Fig). In *Dll*^*212*^*>DrakRNAi*, *rokRNAi* animals, ~50% of the legs had lost distal segments and were considered as truncated while the rest of the legs presented a shortening in length and defects in joint formation (Fig 5I). In prepupal leg discs, the double knockdown of Rok and Drak caused the disruption of several tarsal folds, in a way consistent with the range of joint defects observed in the adult legs (Fig 5B). These results suggest that *rok* and *Drak* have overlapping roles during leg development and joint formation. As Dia is also a downstream target of Rho1, we examined *dia*^*5*^ homozygous mutant clones in the prepupal leg imaginal disc and in the adult leg. Interestingly, removing *dia* function caused strong defects in tarsal fold formation and the lack of joints in the adult legs (Fig 5C and 5J). As some of these leg phenotypes could suggest defects in leg segmentation we have analyzed the expression of *Ser* and the P-D segmentation gene *ap*. In *Dll*^*212*^*>DrakRNAi*, *rokRNAi* prepupal legs we observed that although *ap* expression is unaltered compared to control legs, some tarsal *Ser* bands are disrupted (S6 Fig). Similarly, the localization of Ap and Ser is mostly unaffected in *dia* mutant clones at the exception of a slight downregulation of Ser levels in some mutant cells (S6 Fig).

To further study the role of Rho1 effectors, we examined the result of knocking down the MyoII regulatory light chain, Sqh. We used mutant *sqh* flies carrying a rescue transgene expressing *sqh-GFP* under the control of the *sqh* promoter (*sqh*^*AX3*^*; sqh-GFP* flies). In these flies, a *deGradFP* was expressed in the *ap* domain to degrade the Sqh-GFP fusion protein, and therefore depleting Sqh from the epithelium [47]. As observed in Fig 5D, Sqh-GFP is accumulated in the subapical cell junctions of the leg epithelial cells. Sqh-GFP depletion in the *ap* domain for 24 hrs caused the inhibition of the t4-t5 fold (Fig 5E). Adults of these genotypes have very strong deleterious effects that caused the loss of the t4 segment (Fig 5K). These results are consistent with the requirement of a fine regulation of Rho1 downstream effectors for correct fold and joint formation.

### Activation of Rho1 and its downstream effectors induce fold formation

**Fig 6:**
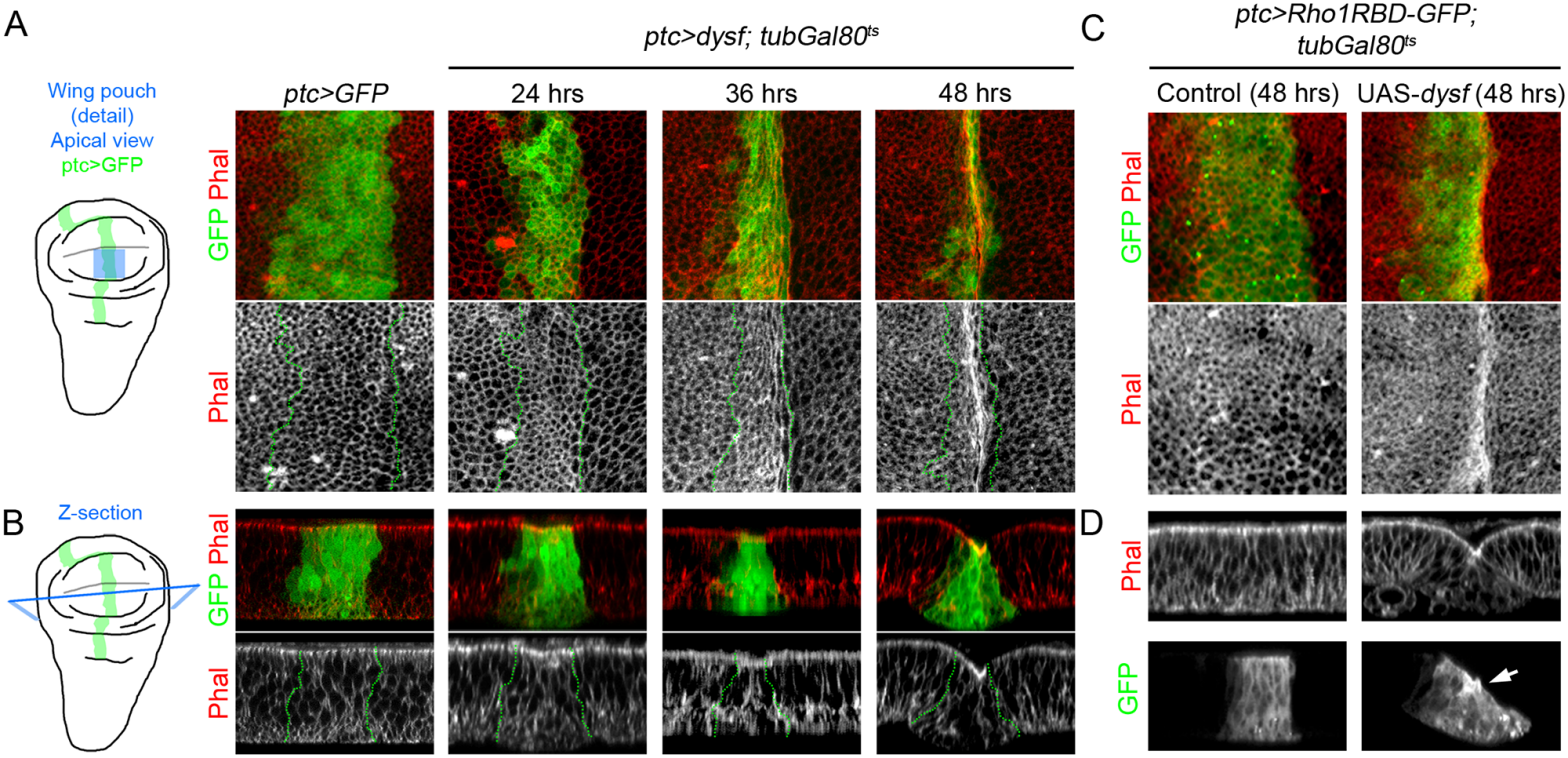
Ectopic expression of *dysf* induces fold formation and Rho1 activation. (A-D) Apical view (A and C) and Z-section (B and D) of a region of the wing pouch (blue square in the schematic wing disc). The *ptc-Gal4* driver is used to express different UAS constructs in a band of cells of the anterior compartment of the wing pouch. Control *ptc>GFP* (A and B) or *ptc*>*Rho1RBD-GFP* (C and D) and different time points of *ptc>dysf* expression, using the *tubulin-Gal80*^*ts*^ system. The *ptc* domain is marked with GFP (green and dotted lines). Phal is in red and separate grey channel where indicated. (A) The *dysf*-expressing cells undergo apical constriction and consequently the *ptc* domain becomes narrower. By 48 hrs, accumulation of F-actin is clearly visible. (B) Z-sections of the genotypes and time points described above. Note the progressive narrowing of the GFP positive cells in their apical region and the formation of a fold that is visible by 48h. (C) The *ptc*>*Rho1RBD-GFP* cells undergo apical constriction and accumulate F-actin after 48 hrs of *dysf* expression. (D) Note that the formation of the ectopic fold in *ptc>dysf* wing pouch is accompanied by an apical accumulation of F-actin and enhanced Rho1 activity in the apical region of the cells that undergo apical constriction.

**Fig 7:**
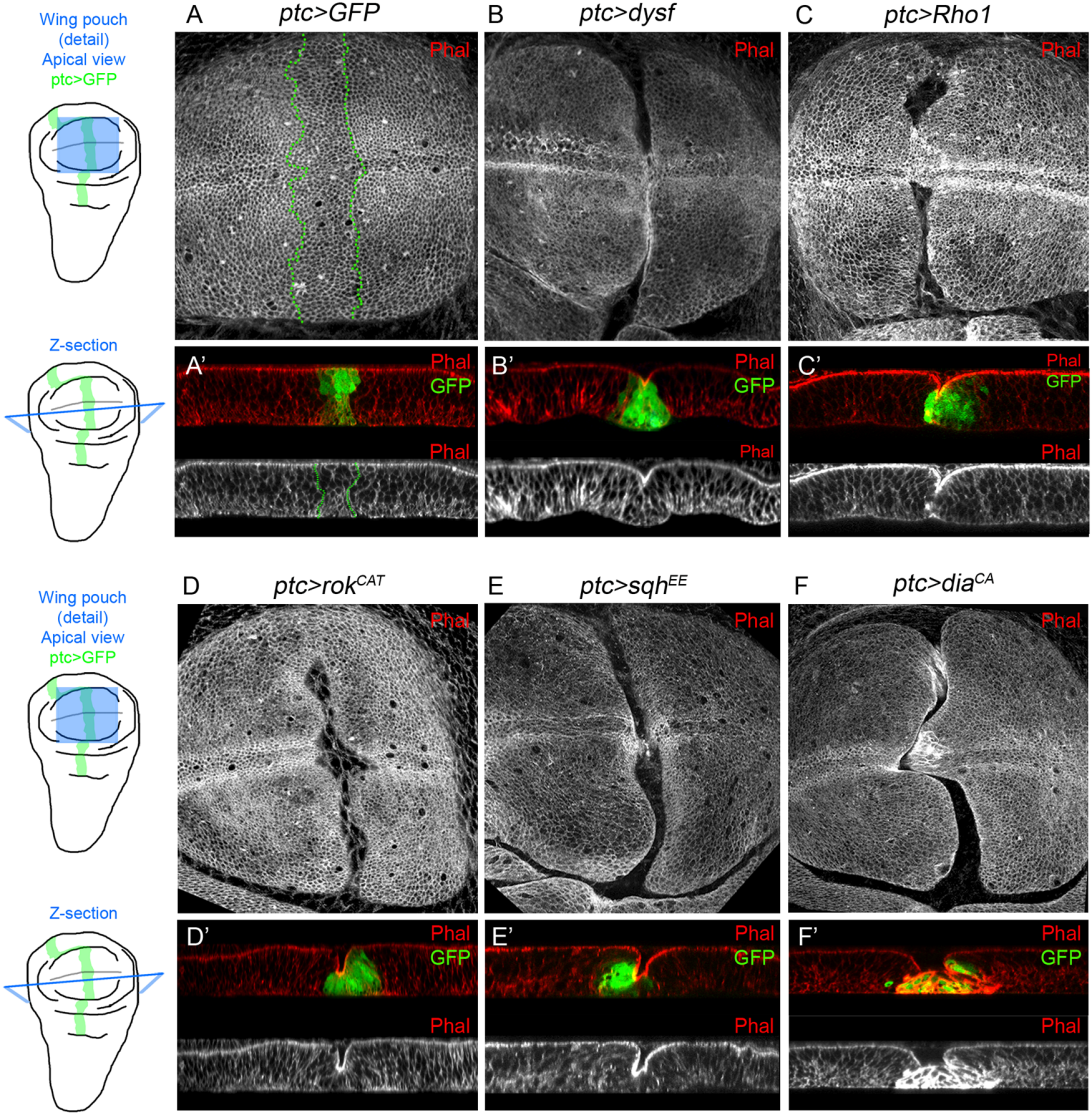
Misexpression of *dysf*, *Rho1* and Rho1 downstream effectors is sufficient to form ectopic folds in the wing disc. (A-F) Apical view of the pouch region of L3 wing discs. The following UAS lines are expressed under control of the *ptc-Gal4* driver (marked with GFP, green and dotted lines in A’-F’): control UAS*-GFP* (A), UAS*-dysf* (B), UAS*-Rho1* (C), UAS*-rok*^*CAT*^ (D), UAS*-sqh*^*EE*^ (E) and UAS*-dia*^*CA*^ (F). UAS expression was restricted with the *tubulin-Gal80*^*ts*^ for 48 hrs in B and 24 hrs in C-F prior to dissection. Z-sections of each genotype are shown in A’-F’. Phal is in red in A’-F’ and separate grey channel in A-F and A’-F’ (lower panels). Note that in the apical views in B-E a cleft is formed in the center of the wing pouch that is caused by apical F-actin accumulation in the *ptc-Gal4* cells that form the fold. In F a cleft is also formed, but the cells in the *ptc-Gal4* domain accumulate F-actin all around their cell membranes, and not restricted to their apices.

Given that Dysf regulates Rho1 activity and the latter contributes to generate the epithelial folds that prefigure the adult tarsal joints, we examined whether the ectopic expression of Rho1 or its downstream effectors could reproduce the formation of ectopic epithelial folds generated by the misexpression of *dysf*. To this end, we performed temporarily restricted gain of function experiments using the *ptc-Gal4* driver in the relatively flat epithelium of the wing disc pouch (Fig 6A and 6B). In order to assess the changes on tissue morphology and cell shape produced by these ectopic expressions we stained the discs with Phalloidin to visualize F-actin cytoskeleton. In the control experiment the expression of *GFP* in the *ptc* domain does not alter wing pouch epithelial morphology or cell shape (Fig 6A and 6B). We previously reported that *dysf* is necessary and sufficient to induce the formation of epithelial folds in the leg [21]. Accordingly, the ectopic expression of *dysf* induces the progressive apical constriction of the cells, and by 48 hrs of *dysf* expression a clear fold in the wing pouch epithelium could be observed, that is accompanied by an accumulation of apical F-actin (Fig 6A and 6B). The cell-autonomous capacity of *dysf* to form ectopic folds was confirmed by generating *dysf* gain of function clones (S7 Fig). Furthermore, Rho1 activity is enhanced at the apical region of the fold-forming cells in a pattern that is reminiscent to what we observed during tarsal fold formation in the leg (compare Fig 6D with Fig 3C). Additionally, MyoII localization (visualized by Zip-GFP) at the adherens junctions is unchanged in *dysf*-induced folds in the wing pouch, a distribution similar to the endogenous leg folds (compare S8 Fig with Fig 2A). Next, we tested whether the ectopic expression of a wild type form of Rho1 is sufficient to fold the wing pouch epithelium, and found that a sharp fold is formed within the *ptc* domain (Fig 7C). Consistent with these results, the expression of a constitutively active form of the Rho1 effector Rok (UAS-*rok*^*CAT*^) also caused the formation of a fold along the *ptc* domain and the apical accumulation of F-actin in the bending cells (Fig 7D). Almost identical results were obtained when we directly activated MyoII contractility by expressing an active form of Sqh, UAS*-sqh*^*EE*^ (Fig 7E). In addition, the expression of a constitutively active version of Dia (UAS-*dia*^*CA*^), also promoted fold formation and increased cortical F-actin (Fig 7F). Together, these ectopic expression experiments confirm the key role of Rho1 and its effectors in the induction of epithelial folds.

## Discussion

In this study we have identified a functional relationship between *dysf*, a Notch-induced transcription factor expressed in concentric rings in the tarsal region of the leg disc and the activation of Rho1 GTPase. We found that Rho1 is active in the epithelial folds that prefigure the formation of the adult leg joints. Rho1 and its effectors, Rok, Drak and Dia are responsible, through the regulation of acto-myosin cytoskeletal dynamics, for the apical constriction and fold formation during tarsal joint morphogenesis. In addition, Dysf, Rho1 and Rho1 effectors are sufficient to induce the formation of epithelial folds when ectopically expressed in a flat epithelium. Surprisingly, our results discard an instructive role for programmed cell death during fold formation and joint morphogenesis. Below we discuss these findings and propose a model for tarsal joint formation.

### Rho1 regulation of tarsal joint formation

Local activation of Rho1 and MyoII can lead to contraction of acto-myosin networks and the induction of epithelial folding through changes in cell shape as occurs in apical constriction [1, 28]. Patterned MyoII activation has been described in multiple morphogenetic processes in *Drosophila*, such as gastrulation, tracheal primordia invagination or eye disc development [48–50]. Although the Rho1 downstream mechanisms that lead to apical constriction are conserved, the upstream signals that trigger Rho1 activity and thus epithelial morphogenesis are tissue specific. For example, during *Drosophila* gastrulation, peak levels of Dorsal activate the *twist* (*twi*) and *snail* (*sna*) genes in the presumptive mesoderm. Subsequently, these transcription factors regulate Rho1 activity through RhoGEF2 localization, driving apical constriction during the invagination process [22, 48, 51, 52]. In this work we describe a similar mechanism of morphogenesis in the leg disc under the control of the Dysf transcription factor.

In the leg disc, the cells that form the tarsal epithelial folds that prefigure the adult joints are characterized by the accumulation of apical F-actin, subsequent apical constriction and a decrease in cell height. A variety of RhoGAPs and RhoGEFs are expressed in the leg disc and are required during tarsal morphogenesis [38]. Our previous results have identified *dysf* as a key player in the formation of tarsal joints regulating the expression of the pro-apoptotic genes *rpr* and *hid* and the Rho1 regulators *RhoGEF2* and *RhoGAP71E* [21, 53]. In this work, we analyzed in detail the role of Rho1 as a regulator of epithelial tarsal morphogenesis that acts downstream of *dysf*. Interestingly, we observed that Rho1, but not MyoII, is preferentially localized in the apical domain of fold forming cells. Furthermore, using a Rho1 activity sensor [41] we found that Rho1 is active in the apical domain and at the level of the adherens junctions of cells that undergo apical constriction and tissue folding. These results suggest a role of Rho1 in activating a pre-existing network of MyoII at the adherens junctions to yield apical constriction. Nevertheless, further investigation would be required to precisely describe the molecular dynamics of apical constriction in this particular model. We also observed Rho1 activation in clusters that may correspond with intracellular membrane vesicles, suggesting a possible role of Rho1 in vesicle trafficking [54]. Recently, RhoGAP68F has been implicated in tarsal fold formation and found to be localized preferentially to Rab4 endosomes inhibiting their return to the cell surface and therefore controlling epithelial remodeling at the presumptive joints [39].

The accumulation of apical F-actin and active Rho1 in these cells is dependent on *dysf*, and therefore on Notch activation. In the absence of *dysf*, Rho1 fails to be correctly localized and activated, most likely due to defects in the expression of *RhoGEFs* and *RhoGAPs* at the presumptive joint domain [21].

Rho1 has a variety of functions during development, including cytoskeletal reorganization and cell adhesion [1]. Our loss of function analysis of Rho1 and its effectors highlight a key role of these genes in the formation of the epithelial folds and adult joints. We observed that permanent Rho1 activity inhibition is deleterious to the tissue and causes epithelial integrity defects and massive cell death. However, short time knockdown of Rho1 activity combined with apoptosis inhibition maintains tissue integrity while disrupting epithelial folding and joint formation. Consistently with the redundant roles that Rok and Drak play in MyoII regulatory light chain phosphorylation [36], we found that Rok and Drak have overlapping roles during joint formation. In addition, we identified Dia as a key player during this process, as in Dia knockdown experiments joint formation is impaired. Regulation of cell adhesion and acto-myosin cytoskeleton contraction are processes both regulated by Rho1. As these processes are coupled, it is not possible to separate the two Rho1 functions. Therefore, our study of Rho1 and Rho1 effectors role during joint formation may be hindered, in part, by loss of epithelial integrity. Interestingly, when ectopically activated in the flat wing pouch epithelium, *Rho1*, *rok*^*CAT*^, *sqh*^*EE*^ and *dia*^*CA*^ induced the accumulation of apical F-actin and the formation of a fold. These effects are very similar to those observed in *dysf* missexpression experiments and during tarsal fold formation in the wild type. In summary, our results further support that patterned Rho1 activity could be a general morphogenetic mechanism to sculpt three-dimensional epithelia.

### Proposed model of tarsal joint formation

The restricted expression pattern of the “leg gap” transcription factors along the P-D axis helps establish the localized expression of the Notch ligands *Dl* and *Ser* [10, 11] (reviewed by [8]). The activation of Notch in concentric rings direct the formation of the leg joints trough the activation of subsidiary transcription factors expressed in every joint, as *d-Ap2*, in proximal joints, as the family of *odd-skipped* genes, or in tarsal joints such as *dysf* ([16, 21, 55] and reviewed in [27]). However, how these transcription factors regulate the cell behavior changes required for the morphogenesis of the joints is mostly unknown. The role of apoptosis as the initial force driving epithelial folding has been recently described [26]. The preferential localization of cell death at the tarsal folds has been proposed to be dependent on the JNK and Dpp pathways and required for joint formation [17, 26]. Importantly, programed cell death also participates in sculpting other morphological structures such as the mouse digits or the grooves separating several *Drosophila* embryo segments ([56] and reviewed in [57]). In this work we have also addressed the relationship between programmed cell death and Rho1 activity during epithelial fold formation. Unexpectedly, we could not find major effects of apoptosis in tarsal joint development, as suppressing cell death in the developing joints is still compatible with fold and joint formation. However, we cannot discard a role of apoptosis in the dynamics of fold formation, regulating the speed or the efficiency of the process; such an effect has been described during both dorsal closure and mammalian neural tube closure, where cell death is not essential for the morphogenesis but accelerates the process [58–60]. Therefore, it is possible that while apoptosis may not be essential for the formation of the joints, as revealed by the lack of phenotype when inhibited, it could contribute to this process during normal development. Instead, our results point to a critical role of regulated Rho1 activity as the leading driver of fold formation. In this scenario, the key cause of fold and joint formation is Dysf modulation of Rho1 activity at presumptive tarsal joints possibly through the regulation of *RhoGEFs* and *RhoGAPs* expression [21]. As Rho1 can induce JNK-dependent cell death in several developmental contexts [45, 61, 62], we propose that the localized presence of apoptotic cells at the tarsal folds may be a consequence of Rho1 activation. In addition, other Rho1-independent mechanisms may exist that could trigger cell death at the presumptive tarsal joints [17]. Altogether, our results identify a link between Notch mediated patterning with Rho1 activity and acto-myosin cytoskeleton dynamics. We propose that this link is exerted by Dysf, a target of Notch that regulates *RhoGEFs/GAPs* expression at the presumptive tarsal joints.

## Materials and methods

### *Drosophila* lines

The following stocks were used: *Dll*^*212*^*-Gal4*, *ap-Gal4*, *ptc-Gal4*, *hhRed ci-Gal4*, *tubGal80*^*ts*^, UAS*-GFP*, UAS-*dysfRNAi* (VDRC #110381), UAS-*dysf* ((Jiang and Crews, 2003) (Bloomington #9592)) UAS-*Rho1*^*N19*^ ([44]; Bloomington #7328), UAS-*Rho1*.*Sph* (Bloomington #58819 (II) and #7334 (III)), UAS-*dia*^*CA*^ ([63]; Bloomington #27616), UAS-*HA*:*rok*^*CAT*^ (kindly provided by J.A. Zallen and described in [41]), UAS-*sqh*^*E20E21*^ (described in [64]), UAS-*flp*, UAS*-αCatRFP* [65], UAS-*dicer*, UAS-*deGradFP* (described in [47]), UAS-*DrakRNAi* (Bloomington #44102), UAS-*rokRNAi* (Bloomington #28797), UAS-*p35* (Bloomington #5073), UAS-*Diap1* (Bloomington #63820 (II) and #6657 (III)), *Drak*^*DEL*^ ([36]), *rok*^*2*^ ([31]), *rok*^*2*^ *Drak*^*DEL*^ (a gift from Franck Pichaud), *sqh*^*AX3*^*; sqh-GFP* ([66]; Bloomington #57144), *dia*^*5*^ ([37]; Bloomington #9138), *dys*^*2*^ and *dys*^*3*^ [67], and the GFP insertions available in the FlyTrap collection (http://flytrap.med.yale.edu/) *Rho1-GFP* (Kyoto Stock Center #110833) and *zip-GFP* (Bloomington #51564). SGMCA was used to monitor F-actin [68]. All the previous lines are described in Flybase and available at Bloomington *Drosophila* Stock Center except otherwise indicated. The *Notch* thermosensitive mutant allele (*N*^*tsa*^) allowed us to knockdown Notch activity when the flies are shifted to the restrictive temperature (29°C) [40]. To assess the activity of Rho1 we used the UAS-*PKNG58AeGFP* line described in [41] under the control of the *Dll*^*212*^*-Gal4* driver. Two different approaches were employed to eliminate cell death: first, we used a transgene that generates miRNAs against the pro-apoptotic genes *rpr*, *hid* and *grim* (UAS-*miRHG*), described in [42] and second, a null mutant allele of the initiator caspase Dronc (*dronc*^*i24*^) [69]. Dysf knockdown with the UAS-*dysf*RNAi line was performed in combination with an UAS-*dicer* to enhance the effect of the RNAi.

### Loss and gain of function experiments

When indicated, prepupae were synchronized to properly compare fold formation phenotypes. White pupae were selected of the given phenotype, incubated for 3 hrs at the required temperature and then dissected and stained following standard procedures. All confocal images were obtained using a Leica LSM510 vertical confocal microscope and were treated using Fiji and Photoshop programs. Quantification of cell death was performed manually in the *hh>*UAS*-miRHG*, UAS-*GFP* experiment to properly distinguish between positive Dcp1 cells that belonged to A or P compartments. To measure Dcp1 levels in *dronc*^*i24*^ homo- or heterozygous mutants we used Fiji to automatize the counting of Z projections of all the cell death present in each leg disc.

To temporarily restrict the activity of the different Gal4 lines we used the *tubulin-Gal80*^*ts*^ system. Briefly, embryos were collected for 24 to 48 hrs, maintained at the restrictive temperature (17°C) and then shifted to the permissive temperature (29°C) for the required time prior to dissection. For the analysis of adult phenotypes in the *ap>Rho1*^*N19*^ and *ap>Rho1*^*N19*^, *miRHG* experiments, larvae were kept at 17°C and once wandering L3 appeared in the walls of the tube, the vials were shifted to 29°C for 48 hrs to ensure strong Gal4 activity during fold formation (late larva through early pupal stages) and then transferred back to 17°C until hatching.

### Clonal analyses

*yw hsflp; dia^5^ FRT^40^*/ *ubiGFP M(2)z FRT^40^*

y *rok*^*2*^ *Drak*^*DEL*^ *FRT*^*19A*^/*tubGal80 hsflp FRT*^*19A*^*; act-Gal4*, *UAS-CD8 GFP/UAS-miRHG*

To generate flies in which the whole leg is mutant we used the following genotypes and a duplication on the Y chromosome that covers the *Rok* gene (*Dp(1;Y)shi+3*, *y+*) (Bloomington #5270).

*yw rok*^*2*^ *FRT*^*19A*^/*ubi-GFP M(1)osp FRT*^*19A*^*; Dll*^*212*^*-Gal4*, UAS*-flp*

For the analysis of Rho1 downstream effectors function in the formation of the joints, we categorized the phenotypes of the different genetic combinations attending to their severity by counting the number of tarsal joints that were affected in each leg. These categories were: no defects, 1 to 2 joints affected, 3 to 4 joints affected and leg truncation.

For the gain of function experiments performed in the wing disc, the *ptc>GFP*, *tubGal80*^*ts*^ line was crossed with the different UAS lines and the progeny maintained at the restrictive temperature (17°C) until shifted to the permissive temperature (29°C) for periods of 24 to 48 hrs before dissection.

For prepupal analysis, *N*^*tsa*^ larvae were grown at 17°C, transferred to 29°C for 72 hrs prior to dissection, and the vials were kept at 29°C to recover adult legs.

### Immunostaining and adult leg preparations

Standard procedures were used to fix and stain prepupal and larval leg and wing imaginal discs. Briefly, larvae and prepupae were dissected in PBS and fixed with 4% paraformaldehyde in PBS for 25 minutes at room temperature. They were blocked in PBS, 1% BSA, 0.3% Triton for 1 hour, incubated with the primary antibody over night at 4°C, washed four times in blocking buffer, and incubated with the appropriate fluorescent secondary antibodies for 1.5 hours at room temperature in the dark. They were then washed and mounted in Vectashield (Cat# H-1000 RRID:AB_2336790). We used anti-Phalloidin (TRITC) (Sigma-Aldrich Cat# P1951 RRID:AB_2315148) to stain the actin cytoskeleton, and TOPRO (Thermo-Fisher Cat# T3605) to stain nuclei. As primary antibodies, we used mouse anti-Dlg (DSHB Cat# 4F3 RRID:AB_528203), rabbit anti-cleaved *Drosophila* Dcp-1 (#9578 Cell Signaling Technology) to mark cell death, rat anti-Ser (a gift from Ken Irvine, Rutgers University, 1/1000) and rabbit anti-Ap [70].

TUNEL analysis was performed using In Situ ‘Cell Death Detection Kit’ (TMR Red) (#12156792 910) and ‘Tunel Dilution Buffer’ (#11966006001) kits, both from Roche.

Rho1RBD-GFP and F-actin relative fluorescence intensity was quantified by measuring the mean intensity of fold and interfold domain cells (Ser positive cells) using Fiji software.

Adult or pharate (in the case of flies that could not hatch) legs of the required phenotypes were collected in 96% ethanol until mounted. We used Hoyer’s mounting medium in a 1:1 proportion with lactic acid (90% MERCK) to preserve the cuticle of the legs [71]. Multiple focal planes of each leg were acquired and then combined using the Helicon Focus program to create a fully focused image of the legs.

## Supporting information

S1 Fig Time course of tarsal fold formation. (A) Apical view of the *ap* domain that encompasses the entire fourth tarsal segment and the cells that would form the fold between t4 and t5 segments (brackets). Expression of *αCatenin-RFP* under the control of the *ap-Gal4* driver is used to visualize cell borders at the level of the adherens junctions in different time points of fold formation: Pre-fold stage, Mid-fold stage and Late fold stage. Underneath each panel is a sagittal section of the t4-t5 region of a wild type prepupal leg disc during joint formation. Phal is used to visualize F-actin cytoskeleton. Note the accumulation of F-actin that starts during Mid-fold and the consequent formation of a fold. The brackets indicate the fold forming cells that undergo apical constriction. (B) Apical view of an *ap>αCatenin-RFP* prepupal leg disc in a *dysf* mutant background (*dysf*^*2*^*/dysf*^*3*^). Underneath is a sagittal view of the t4-t5 region of a *dysf*^*2*^*/dysf*^*3*^ mutant prepupal leg disc. F-actin is visualized by SGMCA (a construct that express the actin-binding region of Moesin coupled to GFP in all the cells), and no accumulation or folding is observed in these discs. Brackets indicate the rows of cells that would form the t4-t5 fold, and that remain unconstricted in *dysf* mutants.
(TIF)

S2 Fig Rho1 activity and Ser pattern in control, *Dll*^*212*^*>miRHG* and *Dll*^*212*^*>dysfRNAi* prepupal leg discs. (A-C) Sagittal view of prepupal leg discs of the following genotypes: *Dll*^*212*^*>Rho1RBD-GFP* (A), *Dll*^*212*^*>Rho1RBD-GFP*, *dysfRNAi* (B) and *Dll*^*212*^*>Rho1RBD-GFP*, *miRHG* (C) stained with Ser antibody to delimitate interfold regions (blue and separate channel). Rho1RBD-GFP is in green and in a separate channel and Phal is used to visualize F-actin (red and separate channel). t2, t3 and t4 indicate tarsal segments.
(TIF)

S3 Fig Time course of active Rho1 localization during tarsal fold formation. Sagittal view of the t4-t5 tarsal region during fold formation at the Pre-fold, Mid-fold and Late fold stage of *Dll*^*212*^*>Rho1RBD-GFP* prepupal leg discs. Rho1RBD-GFP is in green and Phal is in red. Separate grey channels are shown below. Active Rho1 is progressively accumulated, along with F-actin, in the apical region of the fold-forming cells.
(TIF)

S4 Fig Rho1 activity pattern is altered in *N*^*tsa*^ prepupal leg discs. (A) Prepupal leg disc expressing *Dll*^*212*^*>Rho1RBD-GFP* in a *N*^*tsa*^ mutant background. (B, C) Sagittal views of the distal leg epithelium (B) and of a magnification of the putative fold region (C) of the above genotype. Note that the characteristic GFP pattern observed in control discs (*Dll*^*212*^*>Rho1RBD-GFP*, Fig 3A, 3B and 3C) is lost in A, B and C, where GFP levels remains homogeneous throughout the epithelium. Phal is in red and Rho1RBD-GFP in green and in separate channel below. False color is used to enhance contrast in C (right panel).
(TIF)

S5 Fig Cell death inhibition does not impair fold nor joint formation. (A-D) L3 leg discs of control *hh>GFP* (A and C), *hh>miRHG* (B) and *hh>2xDiap1* (D). Posterior compartment is marked by GFP (green in A-D and dotted green line in A’-D’). Dcp1 to visualize cell death is in red in A-D and in separate channel in A’-D’. (E and F) L3 leg disc heterozygous (E) and homozygous (F) for *dronc*^*i24*^ null allele. Both discs are stained for Tunel to visualize nuclear fragmentation (green in E and F and separate channel in E’ and F’). Dcp1 is in red and Phal is in blue. (G and H) Prepupal leg discs heterozygous (G) and homozygous (H) for *dronc*^*i24*^ allele. Note that the tarsal folds remain formed despite decreased levels of cell death in H. Dcp1 is in red and Phal is in green. (I, J) Prepupal leg discs of heterozygous *dronc*^*i24*^ (I), used as control, and *Dll*^*212*^*>miRHG* in a *dronc*^*i24*^ homozygous background (J). Dcp1 is in red and Dlg in green. Note that Dcp1 levels are completely eliminated in J, whereas folds are formed as in the control disc. (K) Prepupal leg disc of *Dll*^*212*^*>p35*. Dcp1 is in red and Phal is in green and separate channel in K’. Note that Dcp1 staining is observed within the epithelium, but the cells do not show the rounded and fragmented morphology typical of apoptosis, while folds are still correctly formed. In all the confocal panels, a Z-stack of all the planes of the Dcp1 and Tunel channels is presented to show the total cell death present in each disc. (L) Quantification of Dcp1 positive cells in A and P compartments of control (*hh>GFP*, n = 9), *hh>miRHG* (n = 12) and *hh>2xDiap1* (n = 12) L3 leg discs. ****p < 0.0001, with Student’s t test, indicating a significant difference from control. ns, non-significant. Error bars represent SEM. Observe that, while Dcp1 levels in the Anterior compartment are comparable within experiments, cell death is significantly reduced in the Posterior compartment upon cell death inhibition either by UAS-*miRHG* or UAS-*Diap1* expression. (M) Quantification of cell death in *dronc*^*i24*^ heterozygous and homozygous L3 leg discs (n = 10 and 11, respectively). ****p < 0.0001, with Student’s t test, indicating a significant difference from control. Error bars represent SEM. (N) Tarsal region of adult legs of different conditions of cell death inhibition. Two representative legs are presented for the *Dll*^*212*^*>p35* genotype. Asterisks indicate defective joint. (O) Quantification of joint defects for the legs shown in N. Severity of the joint phenotypes is assessed by counting the number of legs that presented joint defects: *‘no phenotype’* if no joints are affected; *‘1–2 joints’* when 1 to 2 joints are defective; ‘3–4 joints’ when 3 to 4 joints are affected. The genotypes are as follows: control *Dll*^*212*^*>GFP* (n = 99), *Dll*^*212*^*>dysfRNAi* (n = 47), *Dll*^*212*^*>miRHG* (n = 89), *Dll*^*212*^*>2xDiap1* (n = 155), *dronc*^*i24*^ homozygous mutants (n = 82), *Dll*^*212*^*>miRHG* in a *dronc*^*i24*^ homozygous background (n = 49) and *Dll*^*212*^*>p35* (n = 126). All the experiments were performed at 25°C.
(TIF)

S6 Fig Analysis of joint formation and leg segmentation in loss of function of Rho1 downstream effectors. (A) Phenotypical analysis of the adult legs presented in Fig 5. Legs are grouped as *‘no phenotype’* if no joints are affected; *‘1–2 joints’* when 1 to 2 joints are affected; ‘3–4 joints’ when 3 to 4 joints are affected, and *‘truncated’* when the distal region was totally or partially lost. The genotypes are as follows: *Dll*^*212*^*-Gal4* (n = 55), *Dll*^*212*^*>miRHG* (n = 89), *Dll*^*212*^*>dysfRNAi* (n = 47), *yw rok*^*2*^ *FRT*^*19A*^/*ubi-GFP M(1)osp FRT*^*19A*^*; Dll*^*212*^*>flp* (n = 72), *Dll*^*212*^*>rokRNAi* (n = 86), *Drak*^*Del*^ (n = 100), *Dll*^*212*^*>DrakRNAi* (n = 60) and *Dll*^*212*^*> DrakRNAi*, *rokRNAi* (n = 85). All the experiments were performed at 25°C. (B and C) Adult legs (tarsal region) of the *Dll*^*212*^*>DrakRNAi* and *Dll*^*212*^*>rokRNAi* genotypes, respectively. (D) Prepupal leg disc showing *rok* and *Drak* null mutant clones (marked with GFP, green) induced 72 to 96 hrs AEL that also express UAS-*miRHG* to inhibit cell death. Phal is in red. Note the small size of the clones recovered. (E-G) Prepupal leg disc of *wild type* (E), *Dll*^*212*^*>Drak-RNAi*, *rok-RNAi* (F) and *yw hsflp; dia*^*5*^ *FRT*^*40*^*/ ubiGFP M(2)z FRT*^*40*^ (G) flies. Leg discs are stained for Ser (green in E-G and separate channels in E’-G’) and Ap (blue in E-G and separate channels in E”-G”) to assess patterning of the tarsal region. Note that some Ser bands are partially disrupted by the loss of function of Rho1 effectors, while Ap remains correctly localized.
(TIF)

S7 Fig Ectopic *dysf* is sufficient to form folds autonomously. (A) Apical view of the pouch region of a wing disc where clones of UAS-*dysf* marked positively with GFP were generated. In the center of each clone, a circular accumulation of F-actin could be observed (arrows in A’). (B) Z-section of one of the clones is shown in B, and the formation of a deep fold could be observed. The formation of this fold is accompanied by the accumulation of F-actin in the apices of the cells that form the fold (arrows in B’ and B”). Phal is in red in A and B, in separated channels in A’ and B’ and in false color to enhance contrast in B”. GFP is in green in A and B.
(TIF)

S8 Fig Ectopic *dysf* expression does not alter MyoII localization. (A and B) Apical view of the pouch region of a wild type (A) and a *ptc>dysf; tubulin-Gal80*^*ts*^ (B) wing imaginal disc. *dysf* ectopic expression in B was restricted for 48 hrs prior to dissection. Zip-GFP is shown in green and Phal is in red. (C and D) Z-section of the genotypes presented above. Zip-GFP is in green and in separate channel below and Phal is in red and in separate channel below. Note that Zip-GFP is localized as puncta at the level of the adherens junctions, and that this localization is maintained when an ectopic fold is induced.
(TIF)
