# Supplementary figures and images for: The transcription factor Dysfusion promotes fold and joint morphogenesis through regulation of Rho1
Source: PLoS Genet. 2018 Aug 6;14(8):e1007584. doi: 10.1371/journal.pgen.1007584 (PMC6095628; doi:10.1371/journal.pgen.1007584)

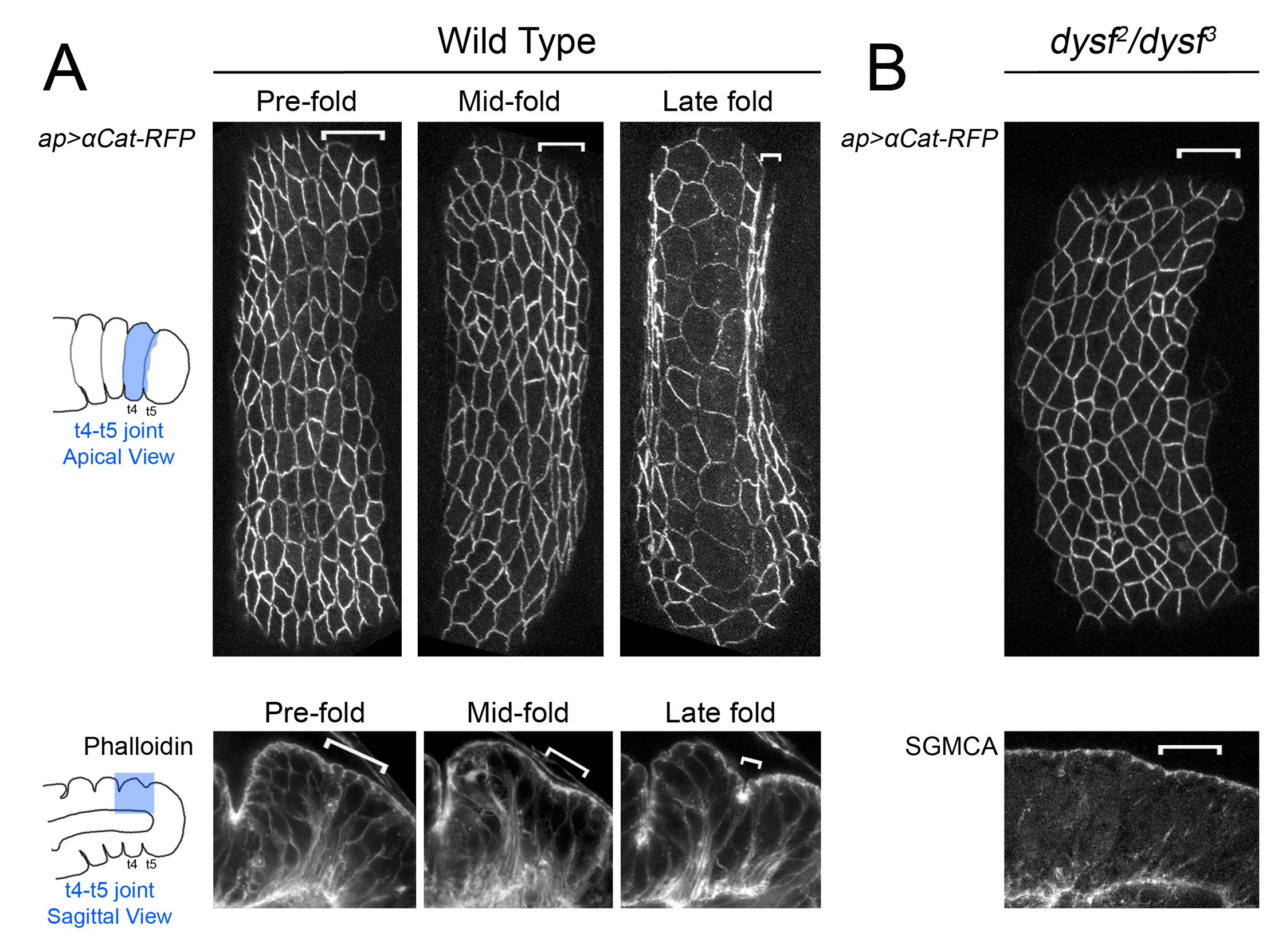

Supplement: S1 Fig — (A) Apical view of the ap domain that encompasses the entire fourth tarsal segment and the cells that would form the fold between t4 and t5 segments (brackets). Expression of αCatenin-RFP under the control of the ap-Gal4 driver is used to visualize cell borders at the level of the adherens junctions in different time points of fold formation: Pre-fold stage, Mid-fold stage and Late fold stage. Underneath each panel is a sagittal section of the t4-t5 region of a wild type prepupal leg disc during joint formation. Phal is used to visualize F-actin cytoskeleton. Note the accumulation of F-actin that starts during Mid-fold and the consequent formation of a fold. The brackets indicate the fold forming cells that undergo apical constriction. (B) Apical view of an ap>αCatenin-RFP prepupal leg disc in a dysf mutant background (dysf2/dysf3). Underneath is a sagittal view of the t4-t5 region of a dysf2/dysf3 mutant prepupal leg disc. F-actin is visualized by SGMCA (a construct that express the actin-binding region of Moesin coupled to GFP in all the cells), and no accumulation or folding is observed in these discs. Brackets indicate the rows of cells that would form the t4-t5 fold, and that remain unconstricted in dysf mutants. (TIF) [file pgen.1007584.s001.tif]

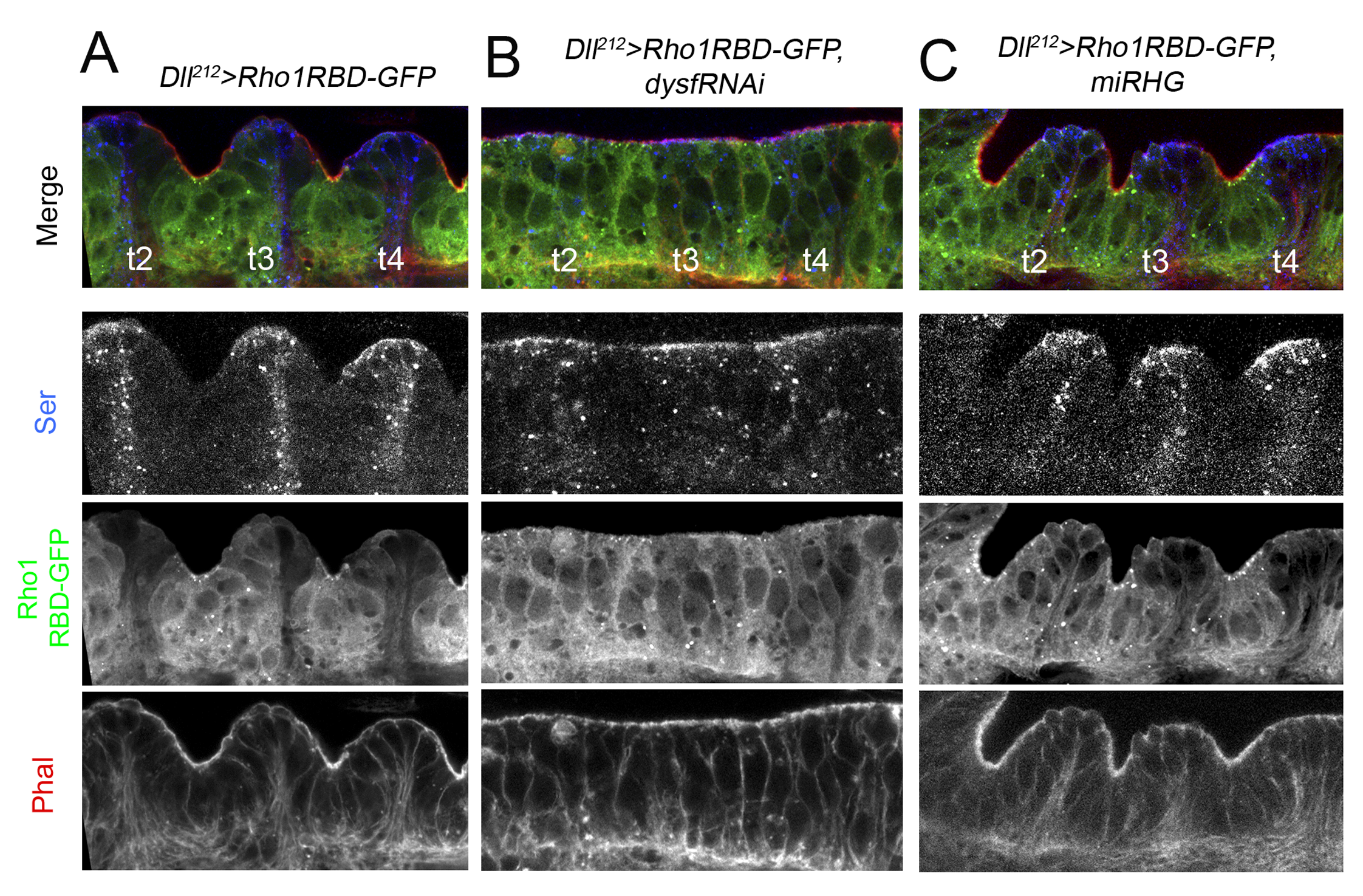

Supplement: S2 Fig — (A-C) Sagittal view of prepupal leg discs of the following genotypes: Dll212>Rho1RBD-GFP (A), Dll212>Rho1RBD-GFP, dysfRNAi (B) and Dll212>Rho1RBD-GFP, miRHG (C) stained with Ser antibody to delimitate interfold regions (blue and separate channel). Rho1RBD-GFP is in green and in a separate channel and Phal is used to visualize F-actin (red and separate channel). t2, t3 and t4 indicate tarsal segments. (TIF) [file pgen.1007584.s002.tif]

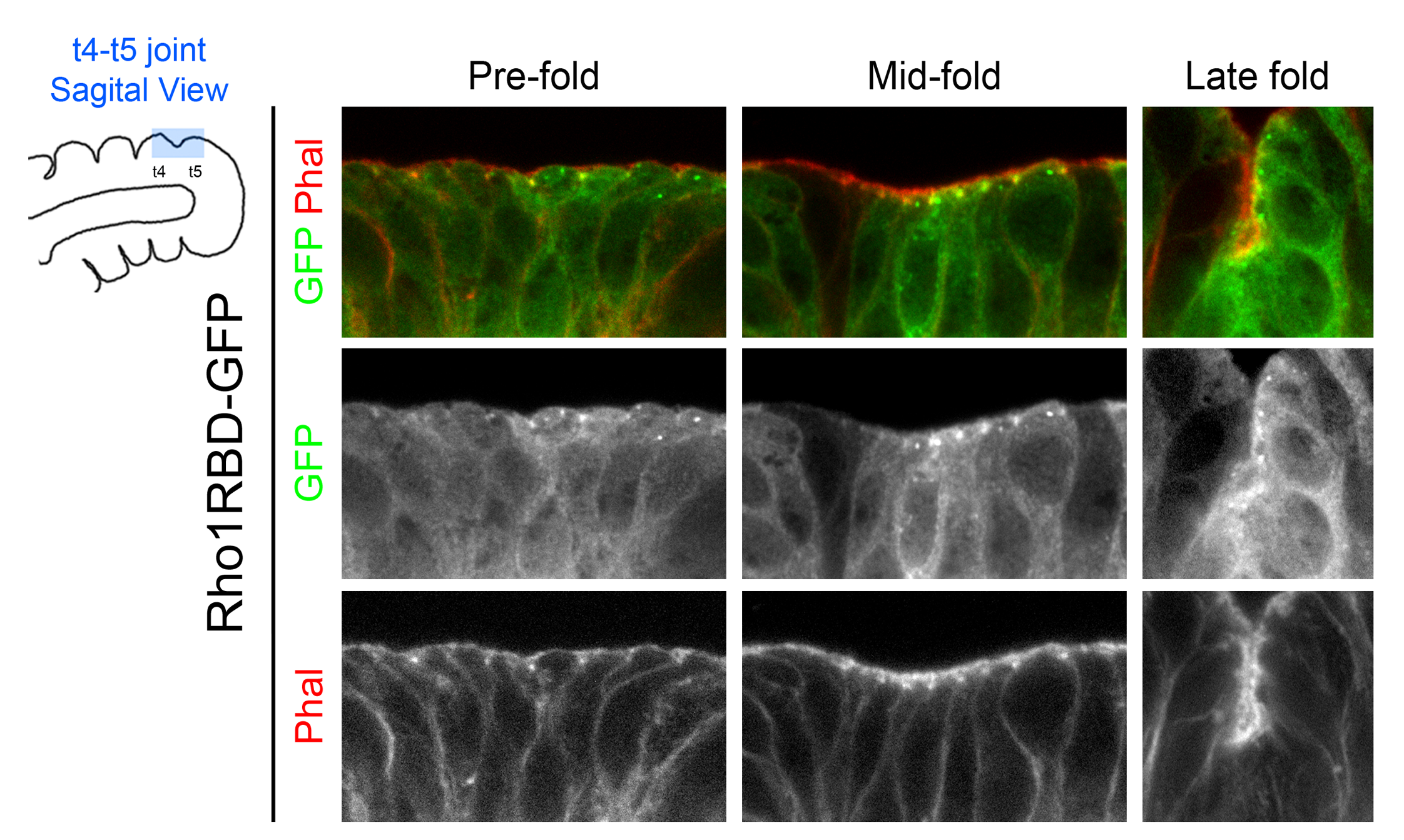

Supplement: S3 Fig — Sagittal view of the t4-t5 tarsal region during fold formation at the Pre-fold, Mid-fold and Late fold stage of Dll212>Rho1RBD-GFP prepupal leg discs. Rho1RBD-GFP is in green and Phal is in red. Separate grey channels are shown below. Active Rho1 is progressively accumulated, along with F-actin, in the apical region of the fold-forming cells. (TIF) [file pgen.1007584.s003.tif]

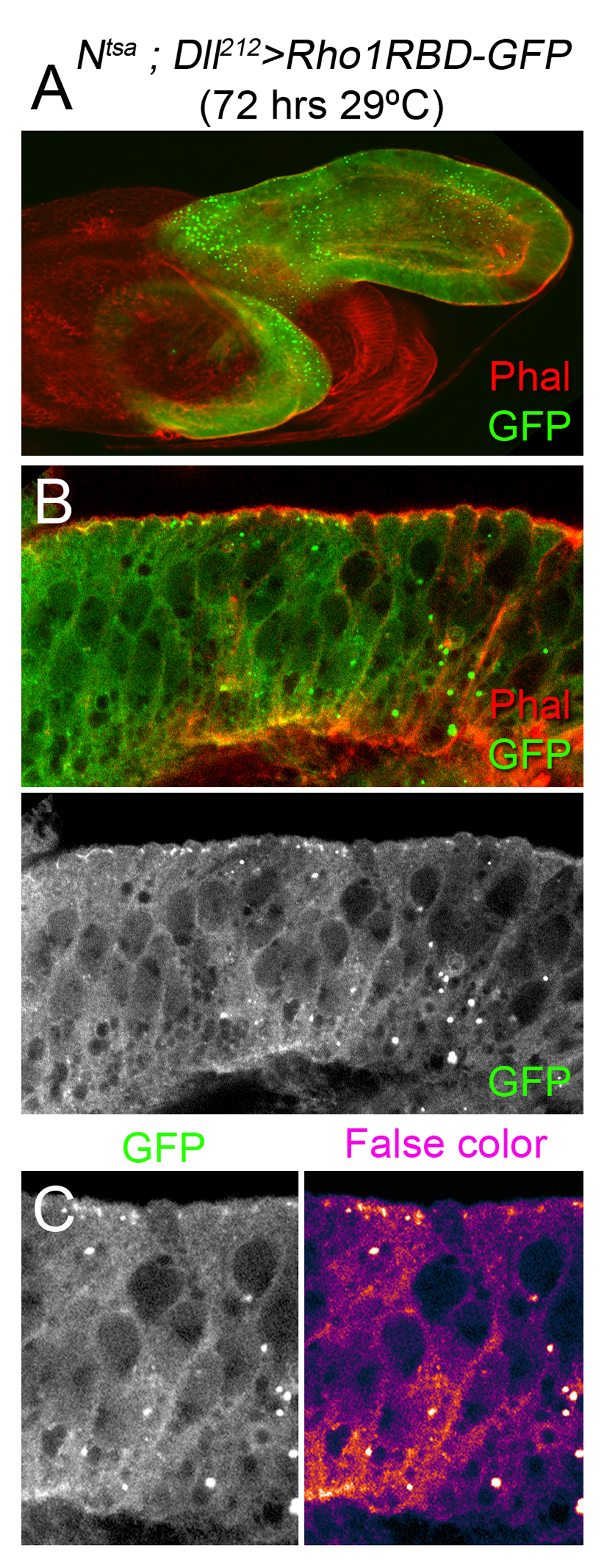

Supplement: S4 Fig — (A) Prepupal leg disc expressing Dll212>Rho1RBD-GFP in a Ntsa mutant background. (B, C) Sagittal views of the distal leg epithelium (B) and of a magnification of the putative fold region (C) of the above genotype. Note that the characteristic GFP pattern observed in control discs (Dll212>Rho1RBD-GFP, Fig 3A, 3B and 3C) is lost in A, B and C, where GFP levels remains homogeneous throughout the epithelium. Phal is in red and Rho1RBD-GFP in green and in separate channel below. False color is used to enhance contrast in C (right panel). (TIF) [file pgen.1007584.s004.tif]

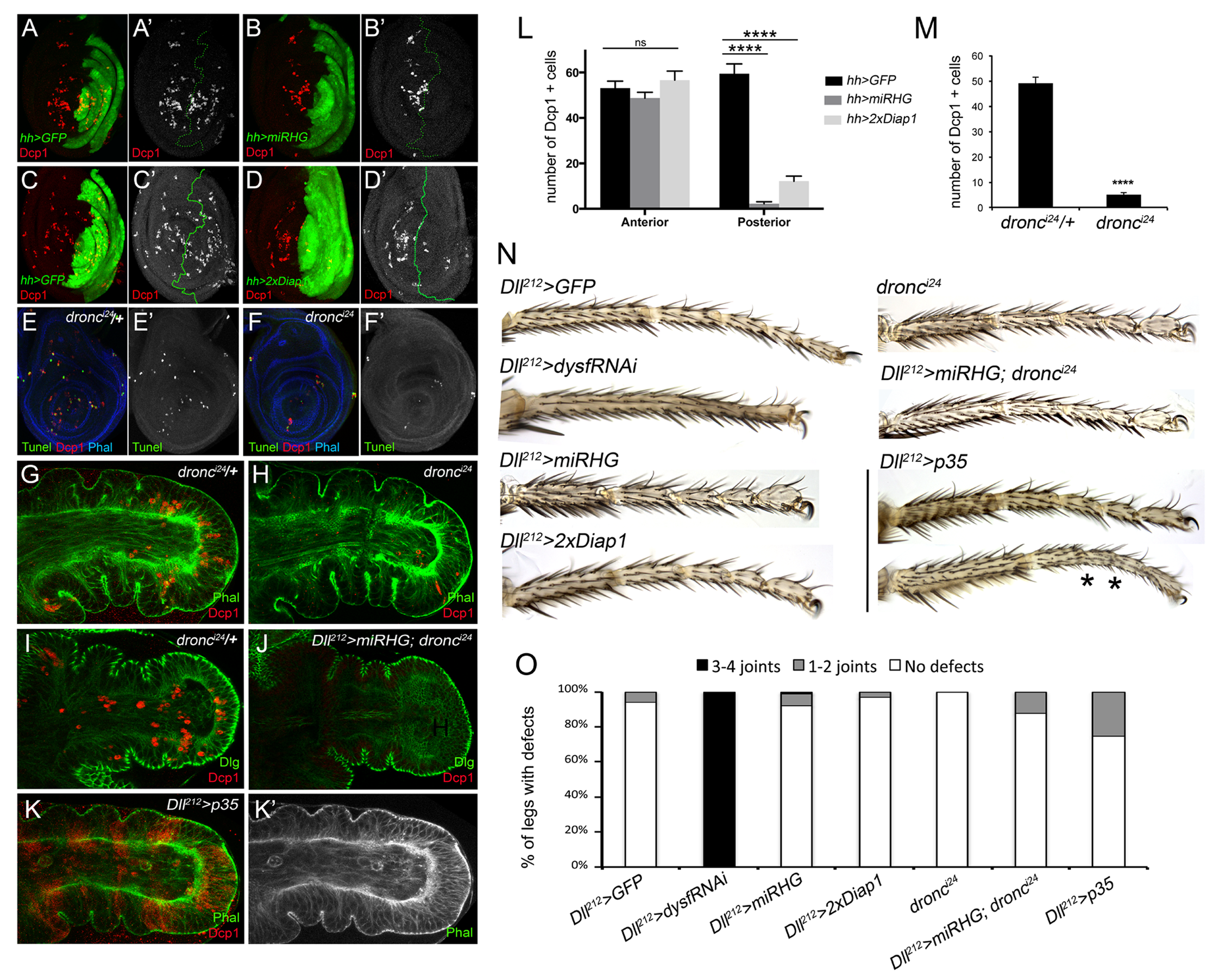

Supplement: S5 Fig — (A-D) L3 leg discs of control hh>GFP (A and C), hh>miRHG (B) and hh>2xDiap1 (D). Posterior compartment is marked by GFP (green in A-D and dotted green line in A’-D’). Dcp1 to visualize cell death is in red in A-D and in separate channel in A’-D’. (E and F) L3 leg disc heterozygous (E) and homozygous (F) for dronci24 null allele. Both discs are stained for Tunel to visualize nuclear fragmentation (green in E and F and separate channel in E’ and F’). Dcp1 is in red and Phal is in blue. (G and H) Prepupal leg discs heterozygous (G) and homozygous (H) for dronci24 allele. Note that the tarsal folds remain formed despite decreased levels of cell death in H. Dcp1 is in red and Phal is in green. (I, J) Prepupal leg discs of heterozygous dronci24 (I), used as control, and Dll212>miRHG in a dronci24 homozygous background (J). Dcp1 is in red and Dlg in green. Note that Dcp1 levels are completely eliminated in J, whereas folds are formed as in the control disc. (K) Prepupal leg disc of Dll212>p35. Dcp1 is in red and Phal is in green and separate channel in K’. Note that Dcp1 staining is observed within the epithelium, but the cells do not show the rounded and fragmented morphology typical of apoptosis, while folds are still correctly formed. In all the confocal panels, a Z-stack of all the planes of the Dcp1 and Tunel channels is presented to show the total cell death present in each disc. (L) Quantification of Dcp1 positive cells in A and P compartments of control (hh>GFP, n = 9), hh>miRHG (n = 12) and hh>2xDiap1 (n = 12) L3 leg discs. ****p < 0.0001, with Student’s t test, indicating a significant difference from control. ns, non-significant. Error bars represent SEM. Observe that, while Dcp1 levels in the Anterior compartment are comparable within experiments, cell death is significantly reduced in the Posterior compartment upon cell death inhibition either by UAS-miRHG or UAS-Diap1 expression. (M) Quantification of cell death in dronci24 heterozygous and homozygous L3 leg [file pgen.1007584.s005.tif]

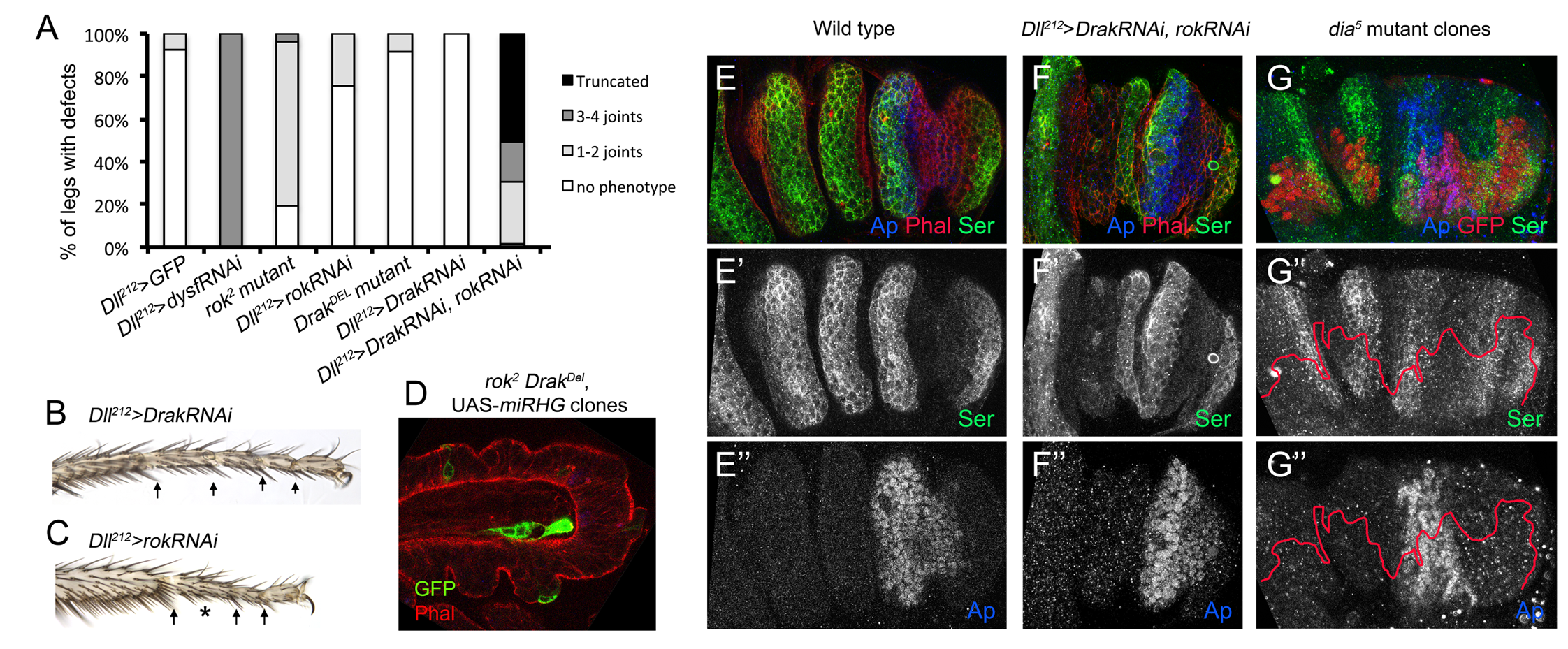

Supplement: S6 Fig — (A) Phenotypical analysis of the adult legs presented in Fig 5. Legs are grouped as ‘no phenotype’ if no joints are affected; ‘1–2 joints’ when 1 to 2 joints are affected; ‘3–4 joints’ when 3 to 4 joints are affected, and ‘truncated’ when the distal region was totally or partially lost. The genotypes are as follows: Dll212-Gal4 (n = 55), Dll212>miRHG (n = 89), Dll212>dysfRNAi (n = 47), yw rok2 FRT19A/ubi-GFP M(1)osp FRT19A; Dll212>flp (n = 72), Dll212>rokRNAi (n = 86), DrakDel (n = 100), Dll212>DrakRNAi (n = 60) and Dll212> DrakRNAi, rokRNAi (n = 85). All the experiments were performed at 25°C. (B and C) Adult legs (tarsal region) of the Dll212>DrakRNAi and Dll212>rokRNAi genotypes, respectively. (D) Prepupal leg disc showing rok and Drak null mutant clones (marked with GFP, green) induced 72 to 96 hrs AEL that also express UAS-miRHG to inhibit cell death. Phal is in red. Note the small size of the clones recovered. (E-G) Prepupal leg disc of wild type (E), Dll212>Drak-RNAi, rok-RNAi (F) and yw hsflp; dia5 FRT40/ ubiGFP M(2)z FRT40 (G) flies. Leg discs are stained for Ser (green in E-G and separate channels in E’-G’) and Ap (blue in E-G and separate channels in E”-G”) to assess patterning of the tarsal region. Note that some Ser bands are partially disrupted by the loss of function of Rho1 effectors, while Ap remains correctly localized. (TIF) [file pgen.1007584.s006.tif]

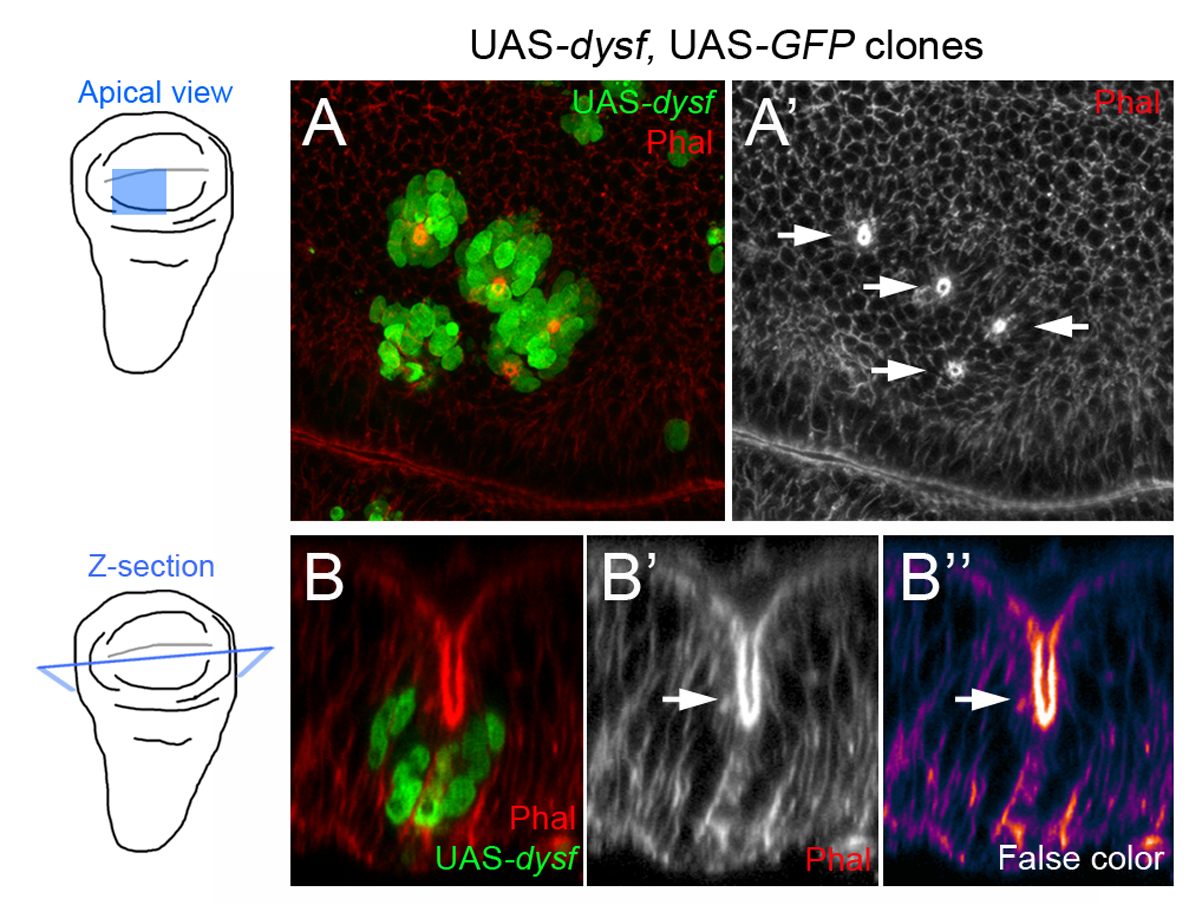

Supplement: S7 Fig — (A) Apical view of the pouch region of a wing disc where clones of UAS-dysf marked positively with GFP were generated. In the center of each clone, a circular accumulation of F-actin could be observed (arrows in A’). (B) Z-section of one of the clones is shown in B, and the formation of a deep fold could be observed. The formation of this fold is accompanied by the accumulation of F-actin in the apices of the cells that form the fold (arrows in B’ and B”). Phal is in red in A and B, in separated channels in A’ and B’ and in false color to enhance contrast in B”. GFP is in green in A and B. (TIF) [file pgen.1007584.s007.tif]

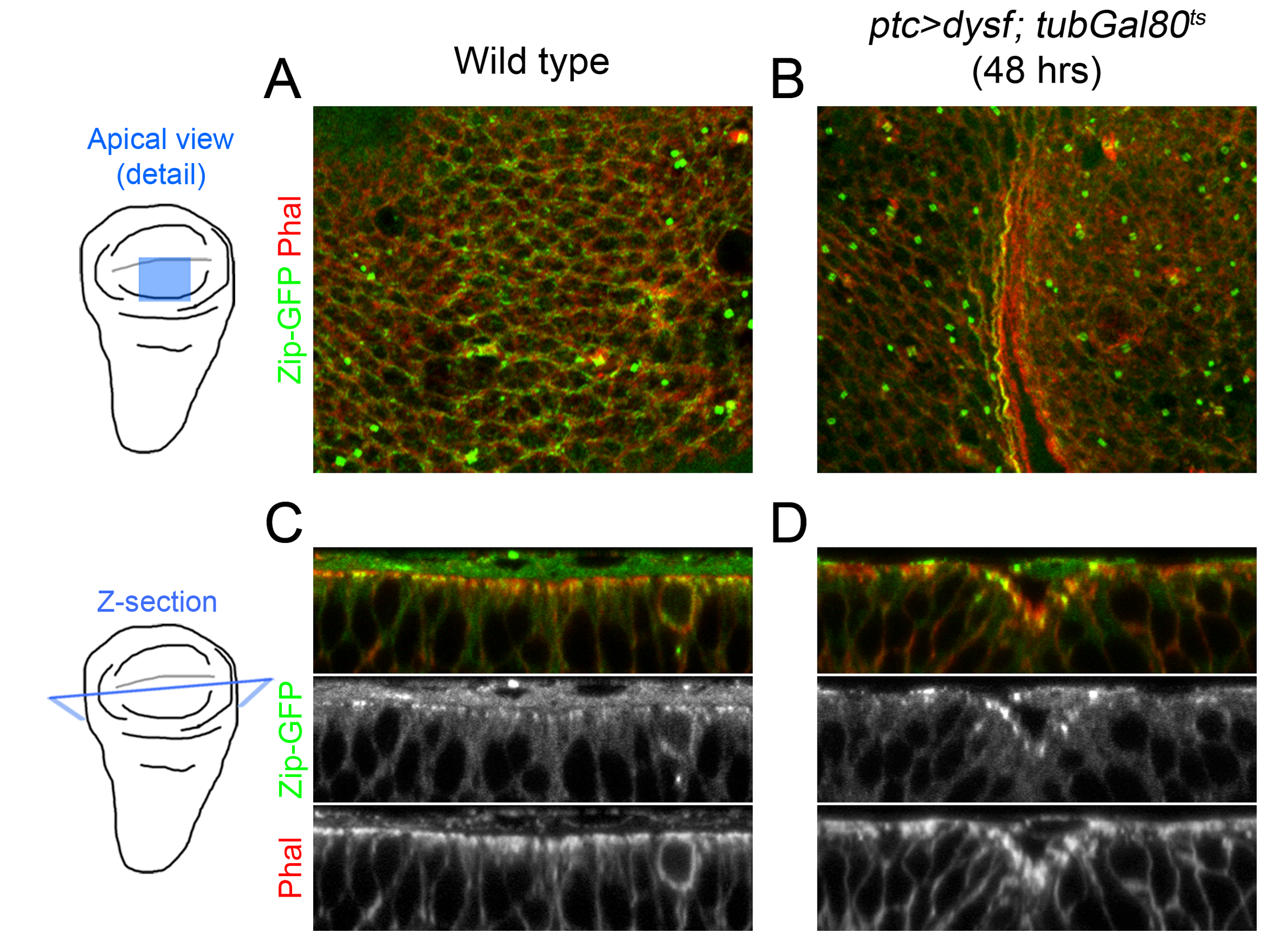

Supplement: S8 Fig — (A and B) Apical view of the pouch region of a wild type (A) and a ptc>dysf; tubulin-Gal80ts (B) wing imaginal disc. dysf ectopic expression in B was restricted for 48 hrs prior to dissection. Zip-GFP is shown in green and Phal is in red. (C and D) Z-section of the genotypes presented above. Zip-GFP is in green and in separate channel below and Phal is in red and in separate channel below. Note that Zip-GFP is localized as puncta at the level of the adherens junctions, and that this localization is maintained when an ectopic fold is induced. (TIF) [file pgen.1007584.s008.tif]
